# Temporal relationship between osteoarthritis and comorbidities: a combined case control and cohort study in the UK primary care setting

**DOI:** 10.1093/rheumatology/keab067

**Published:** 2021-03-01

**Authors:** Subhashisa Swain, Carol Coupland, Christian Mallen, Chang Fu Kuo, Aliya Sarmanova, Sita M A Bierma-Zeinstra, Martin Englund, Daniel Prieto-Alhambra, Michael Doherty, Weiya Zhang

**Affiliations:** 1 Academic Rheumatology, Division of Rheumatology, Orthopaedics and Dermatology; 2 Division of Primary Care, School of Medicine, University of Nottingham, Nottingham; 3 School of Medicine, Keele University, Keele, UK; 4 Division of Rheumatology, Allergy and Immunology, Chang Gung Memorial Hospital, Taoyuan City, Taiwan; 5 Musculoskeletal Research Unit, Bristol Medical School, Translational Health Sciences, University of Bristol, Bristol, UK; 6 Department of General Practice, Department of Orthopaedic Surgery, Erasmus University Medical Center, Rotterdam, The Netherlands; 7 Clinical Epidemiology Unit, Orthopaedics, Department of Clinical Sciences, Lund University, Lund, Sweden; 8 Centre for Statistics in Medicine, Nuffield Department of Orthopaedics, Rheumatology and Musculoskeletal Sciences, University of Oxford, Oxford; 9 Pain Centre and Versus Arthritis, University of Nottingham, Nottingham, UK

**Keywords:** osteoarthritis, comorbidity, multimorbidity, temporal association, burden

## Abstract

**Objective:**

To determine the burden of comorbidities in OA and their temporal relationships in the UK.

**Methods:**

The Clinical Practice Research Datalink (CPRD) GOLD was used to identify people with incident OA and age, gender and practice matched non-OA controls from UK primary care. Controls were assigned the same index date as matched cases (date of OA diagnosis). Associations between OA and 49 individual comorbidities and multimorbidities (two or more comorbidities excluding OA) both before and after OA diagnosis were estimated, adjusting for covariates, using odds ratios (aORs) and hazard ratios (aHRs), respectively.

**Results:**

During 1997–2017, we identified 221 807 incident OA cases and 221 807 matched controls. Of 49 comorbidities examined, 38 were associated with OA both prior to and following the diagnosis of OA and 2 (dementia and systemic lupus erythematosus) were associated with OA only following the diagnosis of OA. People with OA had a higher risk of developing heart failure [aHR 1.63 (95% CI 1.56, 1.71)], dementia [aHR 1.62 (95% CI 1.56, 1.68)], liver diseases [aHR 1.51 (95% CI 1.37, 1.67)], irritable bowel syndrome [aHR 1.51 (95% CI 1.45, 1.58)], gastrointestinal bleeding [aHR 1.49 (95% CI 1.39, 1.59)], 10 musculoskeletal conditions and 25 other conditions following OA diagnosis. The aOR for multimorbidity prior to the index date was 1.71 (95% CI 1.69, 1.74), whereas the aHR for multimorbidity after the index date was 1.29 (95% CI 1.28, 1.30).

**Conclusions:**

People with OA are more likely to have other chronic conditions both before and after the OA diagnosis. Further study on shared aetiology and causality of these associations is needed.

**Rheumatology key messages**

- Peope with OA are more likely to have multimorbidity.
- More comorbidities occur after the diagnosis of OA.
- While musculoskeletal, cardiovascular, gastrointestinal and psychological comorbidities are associated before and after OA diagnosis, dementia and systemic lupus erythematosus are only associated with OA after diagnosis.

## Introduction

Comorbidity is defined as the existence or occurrence of any additional chronic condition during the clinical course of a patient who has the index disease under study [1]. There has been growing interest in identifying comorbidities that may associate with OA, especially since the presence of additional comorbidities may increase disease severity and healthcare utilization and require more complex management guidelines [2]. Our recent systematic review found that 60% of people with OA had one or more other chronic conditions, which was 20% greater than those without OA [3]. However, to date, the range of comorbidities studied is primarily limited to cardiovascular diseases (CVDs), diabetes, depression and chronic obstructive pulmonary disease (COPD) [4–6]. Furthermore, because most studies are cross-sectional and the occurrence of comorbidities after OA diagnosis has not been examined, the temporal and causal associations between them have yet to be established. With the exception of shared risk factors, such as ageing and obesity, little is known about the biological plausibility of concurrence of OA and associated comorbidities [7].

Multimorbidity is a rapidly evolving research area in chronic conditions and primary care and is defined as the presence of two or more chronic conditions in the same individual [8]. To date, no studies are available on the reported associations of multimorbidity or comorbidities with OA, and many possible associated conditions have not been examined. In the UK, widespread use of electronic medical records in general practices captures research-quality information on visits, diagnoses, prescribed medications, management and interventions [9]. The longitudinal nature of the recorded data allows the study of information on consultations and diagnoses at multiple time points. Therefore, using data representative of the UK general population in the Clinical Practice Research Datalink (CPRD), this study aimed to examine the burden of comorbidity and multimorbidity both prior to and following the diagnosis of OA compared with matched controls without OA.

## Methods

We used the CPRD GOLD database for both retrospective (before OA diagnosis) and prospective (after OA diagnosis) analyses. The study involved analyses of anonymised patient-level data and was approved by the Independent Scientific Advisory Committee for Medicines and Healthcare Products Regulatory Agency (MHRA) database research (protocol 19_030R).

### Data source

The CPRD contains primary care electronic medical records and is generalisable to the wider UK population [10]. As of 31 December 2017, CPRD contained data on ∼17.5 million individuals from 736 general practices [10]. Substantial research has provided satisfactory results regarding the validity, representativeness and completeness of the CPRD [12]. More details about the database can be found at https://cprd.com/primary-care.

### Case definition of OA

We used Read codes, which is a standard clinical coding system used in general practice in the UK, to identify people with a diagnosis of incident OA between 1 January 1997 and 31 December 2017. The date of the first recorded diagnosis for OA was used as the index date to separate retrospective and prospective analyses. Inclusion criteria for incident OA cases were at least one recorded physician diagnosis of OA for the hip, knee, ankle/foot, wrist/hand or recorded as ‘unspecified’; age ≥20 years at the index date; having active registration for at least 36 months with the up-to-standard (UTS) practice prior to the index date; and registered at a practice flagged as having acceptable data (determined by CPRD standards).

An existing Read code list for OA (www.keele.ac.uk/mrr) was updated and adapted according to the inclusion and exclusion criteria and screened by two independent general practitioners (GPs) before use. The codes obtained from the website were previously matched with International Classification of Diseases, Tenth Revision codes (Musculoskeletal disorder chapter) [13]. Although not all OA joint codes have been validated, a recent study reported a positive predictive value of almost 80% for Read codes for hip OA in people ≥60 years of age [13].

### Selection of controls

Controls were people registered for at least 36 months with UTS practices and with no record of diagnosed OA, OA-related joint pain or total joint replacement. One control was selected per OA case (i.e. 1:1 matching), matched by year of birth (±2 years), gender, year of first registration and practice. The same index date (i.e. date of first OA diagnosis) as their matched case was used.

#### Definitions and extraction of comorbidities and multimorbidity

We defined comorbidity as the recording of a diagnosis of predefined chronic conditions in individuals of both groups. An extensive list of 49 chronic conditions was prepared from the Quality Outcome Framework (QOF) [15], a list of the US Department of Health and Human Services Initiative on Multiple Chronic Conditions [16] and the Charlson comorbidity index [17]. The list was updated with findings from our systematic review [3] and a previous UK community-based knee pain study [3, 18] by including common and important morbidities not included in the above [19, 20]. We also examined the association of multimorbidity (two or more conditions other than OA) with OA before and after the diagnosis/index date.

The 49 comorbidities in our study were further categorised into eight groups, specifically: musculoskeletal (MSK), respiratory, genitourinary, neuropsychiatric, cancer, circulatory, metabolic/endocrine and gastrointestinal (GI). In addition, nine other conditions were grouped as a ninth ‘other’ category (Supplementary Table S1.1, available at *Rheumatology* online). Wherever required, the codes were further refined after comparing with codes used by other researchers in our department and other sources [21, 22]. Most of the comorbidities listed have been externally validated [12, 23]. A final list of codes was shared with our GP collaborator for input and verification. Finally, the corrected codes were reviewed and agreed upon by the research team. A summary of the disease list with primary Read codes is given in Supplementary Table S1.1, available at *Rheumatology* online.

### Covariates

The whole study period was divided into five observation periods (0–1 year, 0–5 years, 0–10 years, 0–15 years and 0–20 years) before and after the index date. We extracted information on BMI, alcohol use and smoking status at the end of each time. If information on these variables was missing in one time period, it was imputed using the last observation carried forward from the previous time interval (i.e. assuming the value remained unchanged). However, for completely missing information we used multiple imputation with chained equations to generate five imputations per person using the MICE package in R software (R Foundation for Statistical Computing, Vienna, Austria).

BMI (in kg/m^2^) was categorized as underweight (<18.5), normal (18.5–24.9), overweight (25.0–29.9) or obese (≥30.0) [24]. Smoking status was categorised as ex-smoker, current smoker or non-smoker. Alcohol use was grouped into non-user, ex-user, current user 1–9 units/week, current user ≥10 units/week or current user (unknown quantity).

### Statistical methods

For the retrospective analysis, a nested matched (please see above about matching) case–control design was used. The prevalence of a specific comorbidity in OA patients and controls was estimated by calculating the proportions of people diagnosed with the comorbidity during the previous 1, 5, 10, 15 and 20 years (maximum) before the index date out of the total number of cases and controls. This method was used primarily to examine whether longer observation periods would give greater prevalence to assess observational bias [25] and because longer observation periods are often needed to capture the diagnosis of chronic diseases in a consultation-based database [26]. Odds ratios (ORs) and 95% CIs were used to estimate the associations between OA and each comorbidity. Multivariable conditional logistic regression was used to adjust for age, BMI, smoking, alcohol use and multimorbidity count at the index date. Age was adjusted to account for the residual variation due to the group matching (±2 years). We also estimated the total number of comorbidities (none, one, two, three and four or more comorbidities) and the OR for multimorbidity (two or more chronic conditions) during the retrospective time periods.

In the prospective analysis, a cohort study design was used. We assessed incident comorbidity at the earliest date of diagnosis after the index date. Both the OA and matched non-OA cohorts were followed up to 20 years after the index date for each specific comorbidity in people without the comorbidity studied at the index date, namely, people at risk. The follow-up period was censored at the earliest date of comorbidity diagnosis, death, transfer out or end of the study (31 December 2017). The Kaplan–Meir method was used to display the cumulative probability of each comorbidity in people with incident OA and matched controls. Hazard ratios (HRs) and 95% CIs were calculated for each comorbidity separately using a Cox proportional hazards model adjusting for age, gender, BMI, smoking, alcohol use, multimorbidity count at the index date and index year. Age, BMI, smoking status and alcohol use were included as time-varying covariates. The proportional hazard assumption for each comorbidity was examined with log-log plots and Schoenfeld residual tests. We also assessed the incidence and HR of developing multimorbidity (i.e. recording of the new second condition after the index date) in a similar way.

Further analyses were carried out to examine the specific associations with knee, hip, wrist/hand and ankle/foot OA. These were restricted to cases with OA at those joints and their matched controls and associations were estimated both retrospectively and prospectively using the above mentioned methods.

We tested the associations with 49 comorbidities. To address the risk of a higher false discovery rate (FDR) due to ‘multiple significance testing’ [27], the FDR method proposed by Benjamini and Hochberg was used to calculate adjusted *P*-values for both retrospective and prospective analyses [28]. Details of the multiple testing methods is given in Supplementary File 2, page 1, available at *Rheumatology* online. The statistical analyses were performed using Stata version 15 (StataCorp, College Station, TX, USA) and R software version 3.5.

### Sensitivity analysis

As a sensitivity analysis for the prospective study, we re-ran the analysis for each comorbidity restricted to people with OA and matched controls without any comorbidities on or before the index date. This study population can be defined as an ‘at-risk’ group for developing any of the comorbidities of interest. For multimorbidity, the incident date was defined as the date of diagnosis of the second new chronic condition from the index date in an individual. Cox proportional hazard models were used to estimate the HR for each comorbidity adjusted for, smoking status, alcohol use and BMI.

## Results

During the period 1 January 1997 to 31 December 2017, we identified 494 716 incident OA cases [29]. Matched controls could be found for 221 807 cases, with a mean age of 61.1 years at diagnosis (s.d. 13.2) with 58% being women. The mean age of the control population (*n* = 221 807) was 60.9 years (s.d. 13.3) with 58% being women. Table 1 shows the characteristics of the OA cases and matched controls.

**Table 1:** Characteristics of incident OA patients and matched controls at the index date

Characteristics	Incident OA (*n* = 221 807)	Controls (*n* = 221 807)	Unadjusted OR^a^ (95% CI)
Age, mean (s.d.), years	61.05 (13.17)	60.88 (13.31)	
Age (men), mean (s.d.), years	60.71 (12.85)	60.54 (12.97)	
Age (women), mean (s.d.), years	61.30 (13.40)	61.12 (13.55)	
Age (years), *n* (%)			
<40	12 266 (5.53)	13 018 (5.87)	NA
40–49	30 809 (13.89)	31 673 (14.28)	NA
50–59	60 287 (27.18)	59 606 (26.87)	NA
60–69	60 442 (27.25)	59 294 (27.02)	NA
70–79	40 879 (18.43)	40 418 (18.22)	NA
80–89	15 926 (7.18)	15 815 (7.13)	NA
>90	1198 (0.54)	1353 (0.61)	NA
Gender, *n* (%)			
Men	93 895 (42.33)	93 895 (42.33)	NA
Women	127 912 (57.67)	127 912 (57.67)	NA
BMI (kg/m^2^), *n* (%)			
BMI, mean (s.d.)	28.28 (5.62)	26.62 (4.98)	
<18.5 (underweight)	3039 (1.37)	4810 (2.17)	0.85 (0.82 0.90)^*^
18.5–24.9 (normal)	63 547 (28.65)	86 620 (39.06)	Reference
25.0–29.9 (overweight)	82 734 (37.30)	83 013 (37.44)	1.38 (1.36, 1.40)^*^
≥30 (obese)	72 487 (32.68)	47 294 (21.33)	2.14 (2.11, 2.18)^*^
Alcohol consumption (units/week), *n* (%)			
Never	44 117 (19.89)	41 392 (18.67)	Reference
Ex-drinker	6033 (2.72)	5349 (2.41)	1.04 (1.00, 1.08)
Current, 1–9	77 588 (34.98)	80 381 (36.25)	0.89 (0.88, 0.91)^*^
Current, ≥10	43 186 (19.47)	43 226 (19.49)	0.92 (0.91, 0.95)^*^
Current, unknown	50 883 (22.94)	51 409 (23.18)	0.92 (0.91, 0.94)^*^
Smoking status, *n* (%)			
Never smoked	117 536 (52.99)	123 882 (55.86)	Reference
Ex-smoker	62 571 (28.21)	57 668 (26.00)	1.15 (1.14, 1.17)^*^
Current smoker	41 700 (18.80)	40 237 (18.14)	1.10 (1.08, 1.12)^*^
Joints involved, *n* (%)			
Hip	25 091 (11.31)		
Knee	54 841 (24.72)		
Wrist/Hand	13 255 (5.97)		
Ankle/Foot	5311 (2.39)		
Unspecified	158 620 (71.51)		

a Matched by gender, age, practice, and index date. *P-value <0.05. BMI, Body mass index; NA, not applicable.

### Retrospective analysis

Comorbidities prior to OA index date at every 5 years up to 20 years in the OA case and control groups are shown in Table 2. Within the maximum 20 year observational period prior to the index date, 53.1% of cases and 41.8% of controls had multimorbidity.

**Table 2:** Associations between OA (any joint) and comorbidities diagnosed during a maximum period of 20 years prior to the index date (expanded version for every 5 year interval is provided in Supplementary Table S1.3, available at *Rheumatology* online)

Comorbidities	Prevalence		OR (95% CI)	
	OA cases (*n* = 209 601), *n* (%)	Non-OA controls (*n* = 208 799), *n* (%)	Unadjusted	Adjusted^a^
Multimorbidity	117 997 (53.19)	92 899 (41.88)	1.86 (1.83, 1.88)^*^	1.71(1.69, 1.74)^*^
Musculoskeletal				
Ankylosing spondylitis	3258 (1.55)	2158 (1.03)	1.53 (1.45, 1.62)^*^	1.53 (1.44, 1.62)^*^
Back pain	84 092 (40.12)	61 835 (29.61)	1.70 (1.67, 1.72)^*^	1.67 (1.64, 1.69)^*^
Gout	8013 (3.82)	4829 (2.31)	1.69 (1.64, 1.76)^*^	1.52 (1.46, 1.57)^*^
Osteoporosis	6260 (2.98)	4896 (2.34)	1.27 (1.22, 1.32)^*^	1.41 (1.35, 1.47)^*^
Polymyalgia	2226 (1.06)	1243 (0.59)	1.80 (1.68, 1.93)^*^	1.74 (1.62, 1.87)^*^
Rheumatoid arthritis	1956 (0.93)	972 (0.46)	1.97 (1.83, 2.13)^*^	1.95 (1.80, 2.11)^*^
Sjögren’s syndrome	340 (0.16)	202 (0.09)	1.64 (1.38, 1.96)^*^	1.67 (1.39, 2.00)^*^
Systemic lupus erythematosus	122 (0.05)	81 (0.04)	1.49 (1.12, 1.98)	1.54 (1.15, 2.07)^*^
Fibromyalgia	2162 (1.03)	1073 (0.51)	1.95 (1.81, 2.10)^*^	1.89 (1.75, 2.04)^*^
Fatigue	2453 (1.17)	1739 (0.83)	1.42 (1.33, 1.51)^*^	1.42 (1.32, 1.51)^*^
Respiratory				
Asthma	17 029 (8.12)	12 320 (5.9)	1.41 (1.38, 1.45)^*^	1.33 (1.30, 1.37)^*^
COPD	12 642 (6.05)	9296 (4.45)	1.40 (1.37, 1.45)^*^	1.35 (1.31, 1.39)^*^
Genito-urinary				
Chronic kidney disease	8965 (4.27)	7527 (3.6)	1.25 (1.20, 1.29)^*^	1.12 (1.08, 1.16)^*^
Benign prostatic hypertrophy^b^	8436 (4.02)	6365 (3.05)	1.38 (1.32, 1.43)^*^	1.38 (1.33, 1.43)^*^
Renal stones	1923 (0.91)	1567 (0.75)	1.22 (1.14, 1.31)^*^	1.16 (1.09, 1.25)^*^
Neurological/psychiatric				
Stroke	16 158 (7.7)	14 200 (6.8)	1.17 (1.14, 1.20)^*^	1.15 (1.11, 1.19)^*^
Dementia	1068 (0.51)	990 (0.47)	1.07 (0.97, 1.17)	1.09 (0.99, 1.19)
Epilepsy	1376 (0.65)	1125 (0.54)	1.20 (1.11, 1.30)^*^	1.18 (1.08, 1.29)^*^
Multiple sclerosis	348 (0.17)	433 (0.2)	0.79 (0.68, 0.91)^*^	0.80 (0.69, 0.93)^*^
Parkinson’s disease	696 (0.33)	502 (0.24)	1.36 (1.21, 1.53)^*^	1.39 (1.23, 1.57)^*^
Migraine	11 359 (5.41)	8489 (4.06)	1.36 (1.32, 1.39)^*^	1.37 (1.33, 1.41)^*^
Depression	38 417 (18.32)	27 362 (13.1)	1.53 (1.50, 1.56)^*^	1.49 (1.46, 1.52)^*^
Psychosis	398 (0.19)	419 (0.2)	0.94 (0.82, 1.08)	0.86 (0.75, 1.00)
Schizophrenia	1073 (0.51)	1034 (0.49)	1.03 (0.95, 1.12)	0.95 (0.87, 1.04)
Cancer	8972 (4.28)	7984 (3.8)	1.13 (1.09, 1.17)^*^	1.12 (1.09, 1.16)^*^
Circulatory				
Coronary heart disease	18 302 (8.73)	14 262 (6.83)	1.33 (1.30, 1.36)^*^	1.24 (1.21, 1.27)^*^
Arterial/venous	1429 (0.68)	1062 (0.51)	1.34 (1.23, 1.45)^*^	1.29 (1.19, 1.41)^*^
Heart failure	3113 (1.48)	1847 (0.88)	1.72 (1.62, 1.82)^*^	1.52 (1.43, 1.62)^*^
Hypertension	53 659 (25.6)	46 012 (22.03)	1.24 (1.22, 1.26)^*^	1.08 (1.06, 1.10)^*^
Peripheral vascular disease	5539 (2.64)	3906 (1.87)	1.41 (1.35, 1.47)^*^	1.45 (1.39, 1.51)^*^
Metabolic/endocrine				
High cholesterol	26 558 (12.67)	21 865 (10.47)	1.27 (1.24, 1.29)^*^	1.18 (1.16, 1.20)^*^
Diabetes mellitus	16 147 (7.7)	12 656 (6.06)	1.31 (1.27, 1.34)^*^	1.06 (1.03, 1.09)^*^
Hyperthyroid	2047 (0.97)	1843 (0.88)	1.10 (1.03, 1.17)^*^	1.09 (1.02, 1.16)^*^
Hypothyroid	12 276 (5.85)	9793 (4.69)	1.27 (1.23, 1.30)^*^	1.18 (1.15, 1.22)^*^
Digestive				
Gastritis	10 527 (5.02)	7551 (3.61)	1.42 (1.37, 1.46)^*^	1.42 (1.36, 1.45)^*^
Gastroinstestinal bleed	2253 (1.07)	1570 (0.75)	1.43 (1.34, 1.53)^*^	1.42 (1.33, 1.52)^*^
Gall bladder stones	9189 (4.38)	6461 (3.09)	1.44 (1.39, 1.49)^*^	1.27 (1.22, 1.31)^*^
Inflammatory bowel disease	8704 (4.15)	6409 (3.06)	1.38 (1.33, 1.43)^*^	1.36 (1.32, 1.41)^*^
Liver diseases	1029 (0.49)	689 (0.32)	1.47 (1.33, 1.62)^*^	1.42 (1.29, 1.57)^*^
Irritable bowel syndrome	14 335 (6.83)	10 015 (4.79)	1.47 (1.43, 1.51)^*^	1.52 (1.47, 1.56)^*^
Others				
HIV /AIDS	19 315 (9.21)	15 587 (7.46)	1.99 (0.75, 5.32)	2.08 (0.76, 5.75)
Hearing	1313 (0.62)	1136 (0.54)	1.26 (1.24, 1.29)^*^	1.26 (1.23, 1.29)^*^
Psoriasis	4602 (2.19)	3655 (1.75)	1.24 (1.19, 1.30)^*^	1.20 (1.14, 1.25)^*^
Scleroderma	55 (0.02)	54 (0.02)	0.98 (0.67, 1.43)	0.97 (0.65, 1.44)
Sleep disorder	5148 (2.45)	3820 (1.82)	1.43 (1.36, 1.49)^*^	1.35 (1.28, 1.41)^*^
Tuberculosis	417 (0.19)	342 (0.16)	1.21 (1.04, 1.39)	1.25 (1.08, 1.45)
Anaemia	6732 (3.21)	5406 (2.59)	1.25 (1.20, 1.29)^*^	1.25 (1.21, 1.30)^*^
Vision problems	12 179 (5.81)	10 218 (4.89)	1.15 (1.07, 1.25)	1.11 (1.02, 1.21)
Cataract	3258 (1.55)	2158 (1.03)	1.23 (1.19, 1.27)^*^	1.21 (1.17, 1.24)^*^

* *P* < 0.05 adjusted for multiple testing using FDR. COPD, chronic obstructive pulmonary diseases. ^a^Adjusted for age, gender, BMI, smoking, alcohol use, multimorbidity count and index year. ^b^Only for men.

Of the 49 comorbidities studied, significant associations were seen with 40 comorbidities in the past 20 years (Table 2). During this period the adjusted odds ratio (aOR) for multimorbidity prior to OA was 1.71 (95% CI 1.69, 1.74). The strongest associations were seen with rheumatoid arthritis (RA) [aOR 1.95 (95% CI 1.80, 2.11)], fibromyalgia [aOR 1.89 (95% CI 1.75, 2.04)], polymyalgia [aOR 1.74 (95% CI 1.62, 1.87)], back pain [aOR 1.67 (95% CI 1.64, 1.69)] and SS [aOR 1.67 (95% CI 1.39, 2.00)] (Table 2). The prevalence and aORs according to different observational periods prior to the index date are shown in Supplementary Tables S1.2 and S1.3, available at *Rheumatology* online.

Joint-specific associations retrospectively for each comorbidity are given in Supplementary Table S1.4, available at *Rheumatology* online. For hip OA, for 20 years before the index date, leading comorbidities having a positive association were back pain and AS. Leading comorbidities associated with knee OA within 20 years of the index date were fibromyalgia and polymyalgia. For wrist and hand OA, leading associations were seen with gout and back pain. Comorbidities associated with ankle/foot OA within 20 years of the index date were gout and irritable bowel syndrome (IBS) (Supplementary Table S1.4, available at *Rheumatology* online).

### Prospective analysis

**Fig. 1:**
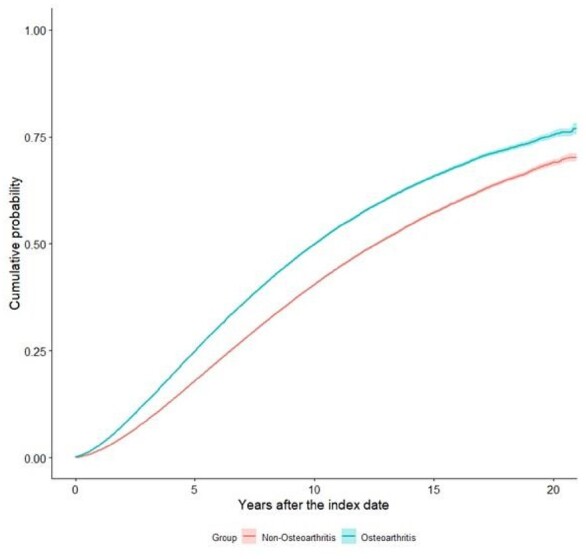
Cumulative probabilities of developing additional multimorbidity in cases with OA and matched non-OA controls irrespective of any comorbidities at the index date

The cumulative probabilities of all comorbidities were higher in the OA group than the control group in each year of follow-up (Supplementary Table S1.5, available at *Rheumatology* online). The adjusted cumulative probabilities of having multimorbidity at 5, 15 and 20 years following the index date were 27.3%, 68.4% and 77.4% in people with incident OA and 19.5%, 42.9% and 70.7% in controls, respectively (Fig. 1). The adjusted HR (aHR) for incident additional multimorbidity was 1.29 (95% CI 1.28, 1.31) in OA cases compared with controls (Table 3).

**Table 3:** HRs and 95% CIs for each comorbidity comparing incident OA (any joint) cases and matched controls for a maximum 20 years of follow-up

Comorbidities	OA cases, incidence/ 1000 p-y	Controls, incidence/1000 p-y		Unadjusted HR (95% CI)	aHR (95% CI)^a^
Multimorbidity	77 695 (6.76)	74 111 (5.12)		1.37 (1.36, 1.39)	1.29 (1.28, 1.30)*
Musculoskeletal					
Ankylosing spondylitis	218 496 (0.8)	217 711 (0.48)		1.63 (1.49, 1.77)	1.44 (1.32, 1.58)*
Back pain	117 392 (42.82)	144 323 (28.99)		1.45 (1.43, 1.47)	1.38 (1.36, 1.41)*
Gout	213 278 (4.46)	214 843 (2.77)		1.63 (1.57, 1.69)	1.41 (1.35, 1.46)*
Osteoporosis	215 723 (5.21)	215 211 (4.47)		1.19 (1.15, 1.23)	1.28 (1.24, 1.32)*
Polymyalgia	219 904 (1.43)	218 863 (0.9)		1.49 (1.40, 1.59)	1.48 (1.39, 1.58)*
Rheumatoid arthritis	219 874 (1.42)	219 077 (0.36)		3.82 (3.50, 4.17)	3.56 (3.26, 3.89)*
Sjögren’s syndrome	221 805 (0.16)	219 902 (0.08)		2.01 (1.64, 2.46)	1.87 (1.52, 2.29)*
Systemic lupus erythematosus	222 027 (0.06)	220 031 (0.02)		2.14 (1.52, 3.01)	1.90 (1.34, 2.69)*
Fibromyalgia	219 834 (1.28)	218 978 (0.37)		3.32 (3.04, 3.63)	2.64 (2.41, 2.89)*
Fatigue	219 556 (1.54)	218 276 (1.06)		1.45 (1.36, 1.54)	1.30 (1.22, 1.38)*
Respiratory					
Asthma	197 561 (3.5)	201 834 (2.53)		1.35 (1.29, 1.40)	1.20 (1.15, 1.25)*
COPD	207 583 (4.13)	209 489 (3.42)		1.22 (1.17, 1.26)	1.18 (1.14, 1.22)*
Genito-urinary					
Chronic kidney disease	212 998 (1.46)	212 652 (1.26)		1.17 (1.15, 1.19)	1.06 (1.04, 1.08)*
Benign prostatic hypertrophy^b^	213 434 (4.01)	213 577 (3.13)		1.27 (1.22, 1.32)	1.27 (1.22, 1.32)*
Renal stones	219 574 (0.74)	217 980 (0.6)		1.25 (1.15, 1.36)	1.10 (1.01, 1.19)
Neurologic/psychiatric					
Stroke	204 629 (8.68)	204 936 (7.26)		1.21 (1.18, 1.24)	1.22 (1.19, 1.26)*
Dementia	221 101 (4.05)	219 204 (3.18)		1.36 (1.32, 1.42)	1.62 (1.56, 1.68)*
Epilepsy	219 002 (0.51)	217 678 (0.37)		1.39 (1.25, 1.54)	1.31 (1.18, 1.46)*
Multiple sclerosis	221 632 (0.09)	219 473 (0.07)		1.18 (0.93, 1.49)	1.09 (0.86, 1.39)
Parkinson’s disease	221 470 (0.79)	219 635 (0.58)		1.41 (1.29, 1.53)	1.46 (1.34, 1.59)*
Migraine	205 856 (2.44)	208 048 (1.74)		1.36 (1.29, 1.43)	1.26 (1.20, 1.33)*
Depression	170 180 (12.86)	182 837 (7.92)		1.58 (1.54, 1.62)	1.43 (1.39, 1.47)*
Psychosis	221 619 (0.19)	219 562 (0.17)		1.10 (0.93, 1.29)	0.94 (0.79, 1.10)
Schizophrenia	220 303 (0.36)	218 301 (0.29)		1.21 (1.07, 1.36)	1.08 (0.96, 1.22)
Cancer	212 110 (9.87)	211 362 (6.72)		1.50 (1.47, 1.54)	1.49 (1.46, 1.53)*
Circulatory					
Coronary heart disease	201 870 (6.32)	204 490 (4.6)		1.35 (1.31, 1.39)	1.22 (1.18, 1.26)*
Arterial/venous	220 674 (1.17)	219 035 (0.84)		1.43 (1.33, 1.53)	1.39 (1.30, 1.49)*
Heart failure	219 010 (2.92)	218 309 (1.69)		1.74 (1.66, 1.83)	1.63 (1.56, 1.71)*
Hypertension	161 900 (23.68)	169 134 (20.58)		1.13 (1.11, 1.15)	1.01 (0.99, 1.03)
Peripheral vascular disease	216 126 (2.93)	215 876 (2.02)		1.45 (1.38, 1.51)	1.36 (1.30, 1.43)*
Metabolic/endocrine					
High cholesterol	194 351 (1.34)	197 519 (1.11)		1.18 (1.16, 1.21)	1.08 (1.05, 1.10)*
Diabetes mellitus	204 495 (11.83)	206 477 (9.05)		1.33 (1.30, 1.36)	1.08 (1.06, 1.11)*
Hyperthyroid	219 061 (0.7)	217 505 (0.57)		1.21 (1.11, 1.32)	1.12 (1.03, 1.22)*
Hypothyroid	208 088 (4.59)	209 156 (3.9)		1.16 (1.12, 1.20)	1.06 (1.02, 1.09)*
Digestive					
Gastritis	207 695 (4.94)	209 676 (3.05)		1.62 (1.57, 1.68)	1.45 (1.40, 1.51)*
Gastrointestinal bleed	219 414 (1.4)	218 162 (0.85)		1.65 (1.54, 1.76)	1.49 (1.39, 1.59)*
Gall bladder stones	209 651 (4.0)	211 412 (2.76)		1.45 (1.40, 1.51)	1.23 (1.18, 1.28)*
Inflammatory bowel disease	211 501 (3.89)	212 175 (2.59)		1.49 (1.45, 1.55)	1.31 (1.26, 1.37)*
Liver disease	220 977 (0.65)	219 294 (0.38)		1.74 (1.58, 1.92)	1.51 (1.37, 1.67)*
Irritable bowel syndrome	222 101 (3.49)	222 145 (2.45)		1.50 (1.44, 1.56)	1.51 (1.45, 1.58)*
Others					
HIV/AIDS	222 161 (<0.001)	220 123 (<0.001)		3.79 (1.23, 11.65)	2.98 (0.95, 9.37)
Hearing	200 102 (12.48)	202 329 (10.92)		1.16 (1.13, 1.19)	1.14 (1.11, 1.16)*
Psoriasis	215 401 (1.3)	214 766 (1.03)		1.23 (1.15, 1.31)	1.14 (1.06, 1.21)*
Scleroderma	222 097 (0.03)	220 060 (0.02)		1.50 (1.05, 21.3)	1.33 (0.93, 1.92)
Sleep disorder	216 765 (3.11)	216 231 (2.06)		1.49 (1.43, 1.56)	1.33 (1.27, 1.39)*
Tuberculosis	220 697 (0.1)	218 804 (0.08)		1.45 (1.16, 1.79)	1.36 (1.09, 1.69)
Anaemia	214 130 (5.62)	213 681 (3.62)		1.57 (1.52, 1.62)	1.42 (1.37, 1.47)*
Vision problems	220 721 (7.62)	218 929 (6.89)		1.12 (1.03, 1.21)	1.09 (1.00, 1.18)
Cataract	222 200 (10.35)	222 215 (9.63)		1.09 (1.07, 1.12)	1.13 (1.10, 1.16)*

p-y, person-years; COPD, chronic obstructive pulmonary diseases. *P < 0.05 adjusted for multiple testing using FDR. ^a^Adjusted for age, gender, BMI, alcohol use, smoking, multimorbidity count and index year. ^b^Only for men.

Except for HIV/AIDS, psychosis, multiple sclerosis, tuberculosis, scleroderma, vision problems, schizophrenia, hypertension and renal stones, the risks of developing each of the other comorbidities were significantly higher in people with OA (Table 3). Patients with OA were >3 times more likely to develop RA [aHR 3.56 (95% CI 3.26, 3.89)] and 2.6 times more likely to develop fibromyalgia [aHR 2.64 (95% CI 2.41, 2.89)]. Besides MSK conditions, people with OA had a higher risk compared with matched controls of developing heart failure [aHR 1.63 (95% CI 1.56, 1.71)], dementia [aHR 1.62 (95% CI 1.56, 1.68)], liver diseases [aHR 1.51 (95% CI 1.37, 1.67)], IBS [aHR 1.51 (95% CI 1.45, 1.58)] and GI bleeding [aHR 1.49 (95% CI 1.39, 1.59)] (Table 3).

Joint-specific results for each comorbidity are given in Table 4. It shows that the risk of being diagnosed with other MSK conditions after an OA diagnosis was higher for all the OA types. People with hip OA had a higher risk of being diagnosed with anaemia and arterial/venous diseases while among people with knee OA the leading comorbidities diagnosed prospectively were GI bleeding and heart failure. After the diagnosis of wrist and hand OA there was an increased risk of sleep disorders and heart failure. In people with ankle/foot OA, the highest risks were for dementia and cancer (Table 4).

**Table 4:** Adjusted^a^ HR and 95% CIs for each comorbidity for a maximum 20 years follow-up, comparing incident OA cases (joint wise) and matched controls irrespective of comorbidities at the index date

Comorbidities	Hip, aHR (95% CI)	Knee, aHR (95% CI)	Wrist/hand, aHR (95% CI)	Ankle/foot, aHR (95% CI)
Additional multimorbidity	1.16 (1.11, 1.21)*	1.24 (1.20, 1.28)*	1.46 (1.36, 1.56)*	1.17 (1.07, 1.29)*
Musculoskeletal				
Ankylosing spondylitis	1.92 (1.47, 2.51)*	1.59 (1.31, 1.93)*	1.82 (1.29, 2.56)*	1.72 (0.98, 2.99)
Back pain	1.36 (1.29, 1.43)*	1.41 (1.36, 1.46)*	1.30 (1.21, 1.39)*	1.38 (1.24, 1.53)*
Gout	1.35 (1.21, 1.51)*	1.42 (1.32, 1.53)*	1.59 (1.34, 1.89)*	1.71 (1.37, 2.13)*
Osteoporosis	1.28 (1.17, 1.40)*	1.37 (1.28, 1.46)*	1.45 (1.27, 1.66)*	1.22 (0.98, 1.52)
Polymyalgia	1.42 (1.18, 1.69)*	1.38 (1.20, 1.58)*	1.67 (1.27, 2.20)*	1.43 (0.90, 2.27)
Rheumatoid arthritis	3.20 (2.40, 4.27)*	2.64 (2.20, 3.17)*	2.27 (1.76, 2.91)*	2.22 (1.28, 3.87)*
Sjörgen’s syndrome	0.95 (0.49, 1.83)	1.61 (0.99, 2.58)	1.72 (0.86, 3.45)	1.72 (0.34, 8.63)
Systemic lupus erythematosus	1.38 (0.58, 3.31)	1.60 (0.78, 3.27)	1.39 (0.38, 5.04)	–
Fibromyalgia	2.32 (1.69, 3.19)*	2.32 (1.88, 2.86)*	1.68 (1.24, 2.28)*	1.68 (0.93, 3.05)
Fatigue	1.42 (1.18, 1.72)*	1.32 (1.15, 1.50)*	1.17 (0.92, 1.50)	1.10 (0.74, 1.64)
Respiratory				
Asthma	1.05 (0.91, 1.20)	1.16 (1.07, 1.28)*	1.25 (1.05, 1.49)	1.30 (0.99, 1.71)
COPD	1.24 (1.12, 1.38)*	1.15 (1.07, 1.24)*	1.13 (0.95, 1.35)	0.99 (0.77, 1.25)
Genito-urinary				
Chronic Kidney Disease	1.14 (1.08, 1.20)*	1.12 (1.07, 1.17)*	1.25 (1.13, 1.38)*	1.23 (1.07, 1.41)*
Benign prostatic hypertrophy^b^	1.27 (1.14, 1.42)*	1.42 (1.32, 1.53)*	1.22 (1.01, 1.47)	1.30 (1.04, 1.62)
Renal stone	1.29 (1.01, 1.65)	1.30 (1.10, 1.54)	0.99 (0.69, 1.41)	1.29 (0.73, 2.31)
Neurologic/psychiatric				
Stroke	1.21 (1.13, 1.31)*	1.24 (1.18, 1.31)*	1.15 (1.02, 1.30)	1.23 (1.04, 1.45)
Dementia	1.66 (1.51, 1.84)*	1.72 (1.60, 1.85)*	1.89 (1.57, 2.28)*	1.95 (1.49, 2.55)*
Epilepsy	1.58 (1.17, 2.12)	1.41 (1.13, 1.74)	1.34 (0.81, 2.19)	1.07 (0.57, 2.01)
Multiple sclerosis	2.18 (1.08, 4.36)	1.05 (0.61, 1.80)	0.82 (0.25, 2.74)	1.33 (0.38, 4.69)
Parkinson’s disease	1.68 (1.34, 2.12)*	1.69 (1.43, 1.99)*	1.25 (0.81, 1.94)	1.83 (1.04, 3.20)
Migraine	1.06 (0.89, 1.25)	1.23 (1.09, 1.37)*	1.27 (2.05, 2.54)	1.25 (0.93, 1.69)
Depression	1.43 (1.33, 1.54)*	1.44 (1.36, 1.51)*	1.36 (1.22, 1.51)*	1.57 (1.34, 1.85)*
Psychosis	0.94 (0.57, 1.55)	0.99 (0.68, 1.43)	1.23 (0.53, 2.83)	0.78 (0.25, 2.44)
Schizophrenia	1.26 (0.87, 1.84)	0.96 (0.74, 1.24)	0.77 (0.42, 1.42)	0.91 (0.42, 1.97)
Cancer	1.60 (1.49, 1.72)*	1.59 (1.51, 1.67)*	1.46 (1.30, 1.63)*	1.65 (1.40, 1.94)*
Circulatory				
Coronary heart disease	1.29 (1.17, 1.41)*	1.30 (1.22, 1.39)*	1.32 (1.14, 1.53)*	1.09 (0.89, 1.34)
Arterial/venous	1.71 (1.42, 2.07)*	1.54 (1.33, 1.77)*	0.93 (0.64, 1.35)	1.64 (1.01, 2.67)
Heart failure	1.64 (1.45, 1.86)*	1.82 (1.66, 2.00)*	1.58 (1.24, 1.99)*	1.36 (0.97, 1.90)
Hypertension	1.05 (0.99, 1.11)	1.04 (1.01, 1.08)	1.08 (0.99, 1.17)	1.01 (0.91, 1.13)
Peripheral vascular disease	1.52 (1.34, 1.73)*	1.41 (1.29, 1.55)*	1.46 (1.19, 1.79)*	1.42 (1.05, 1.93)
Metabolic/endocrine				
High cholesterol	0.97 (0.91, 1.04)	1.08 (1.03, 1.12)*	1.09 (0.99, 1.19)	1.16 (1.01, 1.33)
Diabetes mellitus	1.07 (1.00, 1.15)	1.19 (1.14, 1.25)*	1.24 (1.11, 1.38)*	1.12 (0.97, 1.30)
Hyperthyroid	1.02 (0.79, 1.34)	1.04 (0.86, 1.27)	1.52 (1.04, 2.22)	1.07 (0.62, 1.86)
Hypothyroid	1.02 (0.92, 1.14)	0.96 (0.89, 1.04)	1.16 (0.99, 1.34)	1.14 (0.91, 1.42)
Digestive				
Gastritis	1.57 (1.41, 1.75)*	1.51 (1.40, 1.63)*	1.31 (1.12, 1.53)*	1.39 (1.11, 1.74)*
Gastrointestinal bleed	1.62 (1.34, 1.96)*	1.97 (1.71, 2.26)*	1.28 (0.94, 1.74)	1.52 (1.00, 2.30)
Gall bladder stones	1.33 (1.19, 1.50)*	1.31 (1.20, 1.42)*	1.45 (1.23, 1.70)*	1.13 (0.88, 1.46)
Inflammatory bowel disease	1.41 (1.25, 1.59)*	1.41 (1.29, 1.53)*	1.33 (1.12, 1.58)*	1.62 (1.26, 2.08)*
Liver disease	1.48 (1.09, 2.02)*	1.64 (1.33, 2.00)*	1.38 (0.85, 2.21)	1.49 (0.82, 2.72)
Irritable bowel syndrome	1.26 (1.06, 1.49)*	1.50 (1.33, 1.69)*	1.67 (1.36, 2.04)*	1.50 (1.09, 2.09)*
Others				
Hearing	1.17 (1.10, 1.25)*	1.19 (1.15, 1.25)*	1.23 (1.11, 1.35)*	1.37 (1.19, 1.57)*
Psoriasis	1.09 (0.89, 1.33)	1.05 (0.91, 1.20)	1.12 (0.85, 1.47)	0.97 (0.64, 1.48)
Scleroderma	1.23 (0.47, 3.24)	1.31 (0.54, 3.22)	0.96 (0.24, 3.82)	–
Sleep disorder	1.35 (1.19, 1.54)*	1.39 (1.27, 1.52)*	1.66 (1.35, 2.03)*	1.39 (1.05, 1.86)
Tuberculosis	1.58 (0.68, 3.66)	1.36 (0.85, 2.19)	2.55 (0.99, 6.54)	0.87 (0.24, 3.12)
Anaemia	1.74 (1.59, 1.92)*	1.61 (1.51, 1.72)*	1.33 (1.14, 1.55)*	1.55 (1.25, 1.92)*
Vision problems	1.11 (0.87, 1.40)	1.09 (0.93, 1.29)	1.39 (0.93, 2.09)	1.37 (0.76, 2.48)
Cataract	1.16 (1.07, 1.26)*	1.15 (1.09, 1.22)*	1.27 (1.13, 1.42)*	1.12 (0.92, 1.37)

* *P* < 0.05 adjusted for multiple testing using FDR; COPD, chronic obstructive pulmonary diseases. ^a^Adjusted for age, sex, BMI, alcohol use, smoking, multimorbidity count and index date. ^b^Only for men.

**Fig. 2:**
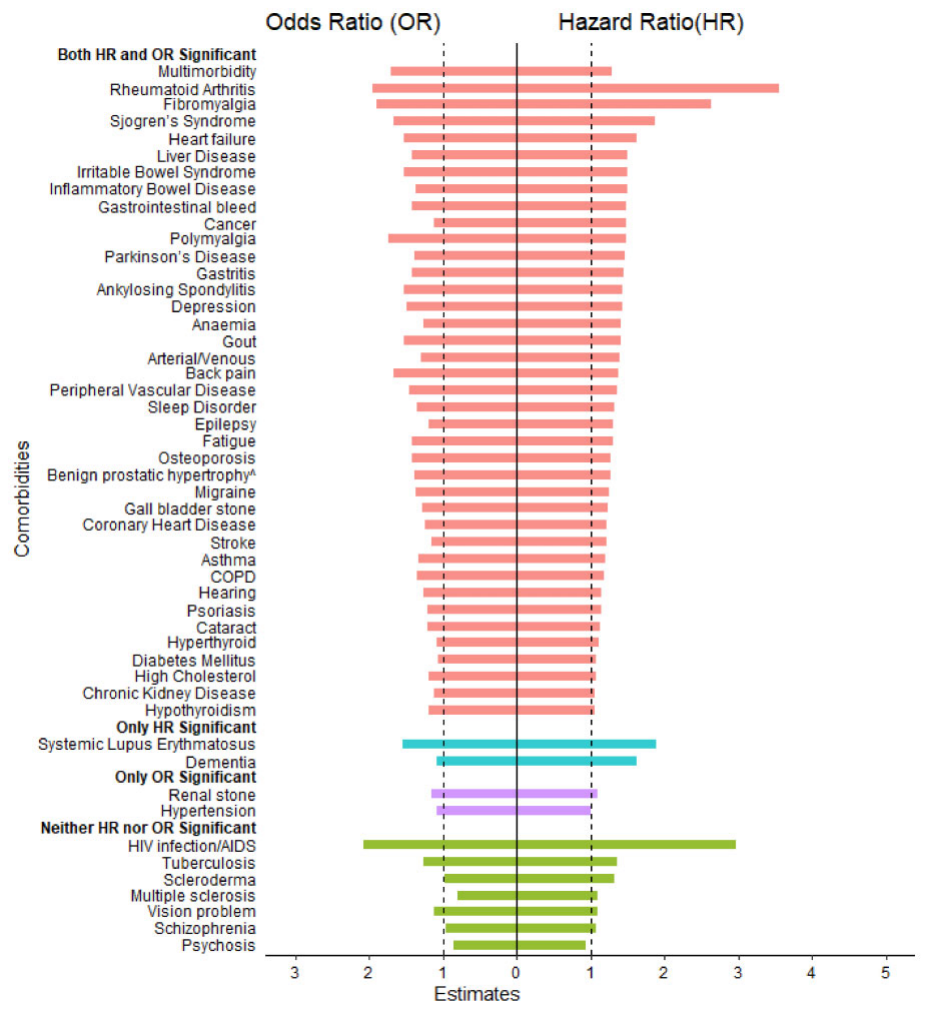
Comparison of aORs and aHRs for comorbidities in OA for a maximum 20 years observation period (before and after index date) Red: both HR and OR significant; blue: only HR significant; purple: only OR significant; green: neither HR nor OR significant. Dashed black line represents statistical significance level at OR or HR = 1. Significant at *P* < 0.05 adjusted for multiple testing using FDR. Both the estimates were adjusted for age, sex, BMI, alcohol use, smoking, multimorbidity count at the index date.

A comparison of aORs and aHRs found that 38 conditions had significant associations with OA both retrospectively and prospectively (Fig. 2). Dementia and systemic lupus erythematosus (SLE) only had a significant association with OA prospectively and hypertension and renal stones only had a significant association with OA retrospectively.

### Sensitivity analysis

The results from sensitivity analyses among OA patients and controls without any comorbidities at the index date showed significant prospective associations for 25 conditions. Comorbidities with the strongest prospective associations were fibromyalgia, RA, liver diseases, sleep problems and GI bleeding. The adjusted risk of developing multimorbidity was 1.34 times greater (95% CI 1.28, 1.41) compared with controls. For more details on the sensitivity analysis see Supplementary File 2, available at *Rheumatology* online.

## Discussion

This study estimated the burden of comorbidities prior to the diagnosis of OA and the risk of developing comorbidities following the diagnosis of OA using a nationally representative large UK primary care database. The key findings are that people diagnosed with OA are significantly more likely to have multimorbidity both prior to and following the diagnosis of OA; while MSK, GI, cardiovascular and psychological conditions were associated with OA in both directions, dementia and SLE were only associated with OA after its diagnosis. 

### Associations in both retrospective and prospective analyses

In this study, OA was found to be associated with large numbers of conditions. This is the first-ever study to examine the association of OA with a large number of conditions in the same primary care cohort. Multimorbidity associations with OA before and after the diagnosis reveal the important role of MSK conditions. Both multimorbidity and OA have positive relationships with ageing. Multiple shared risk factors such as obesity, physical inactivity, medication use and the possible role of inflammation in multimorbidity might lead to OA, and vice versa [30, 31]. Age-related changes in widespread structural components such as collagen and reduced reparative potential with age may also play a role in development of ‘degenerative diseases’ in multiple tissues and systems [32].

Associations of OA with some of the identified MSK comorbidities in this study agree with previous studies [33], such as for RA [34]. The bidirectional associations with discrete chronic pain–related conditions such as fibromyalgia, back pain and IBS could result from shared non-restorative sleep and central pain sensitization, which causes a decreased pain threshold and exacerbation of other causes of pain [35, 36]. The association of OA with gout was stronger before the diagnosis of OA than after, and this might be explained in part by the ‘amplification loop’ of cartilage damage enhancing urate crystal deposition and urate crystals causing cartilage damage [37]. We also found the risk of osteoporosis following a diagnosis of OA was higher than the risk before the diagnosis of OA, but the evidence of their association remains speculative and controversial [38]. Care must be taken in interpreting these associations, especially where joint pain is the reason for the consultation, since GP diagnoses are predominantly clinical and not pathological. Also, although characteristics of these various MSK conditions differ, there is still the possibility of misdiagnosis, especially for atypical cases.

Cardiovascular diseases (CVDs) such as coronary heart disease and heart failure [39], stroke [3, 40], peripheral vascular disease (PVD) [41] and diabetes [42] are well known to associate with OA. We found prospective risks of developing diabetes, PVD and heart failure were greater in OA compared with the risk of developing OA in people with these conditions. This indicates the possible role of obesity and hypercholesterolaemia among people with CVD in causing OA and possibly the effect of non-steriodal anti inflammatory drugs (NSAIDs) use in people with OA in developing CVD [43]. So screening for metabolic syndrome and CVDs should be considered in people presenting with OA [44].

Even though depression and OA had a significant bidirectional association, a higher risk of depression was seen in people following the diagnosis of OA. A similar finding was seen with sleep disorders. Depression and non-restorative sleep are well recognized to associate with chronic pain experience in OA [5]. Low affect and non-restorative sleep can reduce pain inhibition and cause central sensitization, and equally, chronic pain and reduced participation can cause mood disturbance [45].

The risks of developing gastritis, GI bleeding, liver diseases and gall bladder stones in OA were high compared with developing OA in these conditions. GI disorders are known comorbidities in OA resulting from NSAID use [47]. However, recording of incident OA in people with these conditions could result from self-medication for OA pain before presenting to the general practitioner and being diagnosed with OA (i.e. protopathic bias). Interestingly, the risk of OA in liver cirrhosis is reported to be high, but the reverse relationship has yet to be established [48].

Other comorbidities with significant bidirectional associations with OA were respiratory, hypothyroidism and neurological conditions such as Parkinson’s disease, epilepsy and migraine. Thyroid disease, epilepsy, migraine and respiratory illness may have an earlier age of onset than OA, which could have led to their early recording in the database prior to OA. Also, these comorbidities could be mediated through systemic inflammation, medication use or other comorbidities in OA. The four other conditions with bidirectional positive associations in this study were anaemia, BPH, cancer and hearing problems, which have all been reported before [49, 50]. The use of NSAIDs in people with OA/RA was found to reduce haemoglobin levels in one previous study [51]. Release of inflammatory substances has been linked with sensorineural hearing loss [52], BPH [53], cataracts [54] and cancer [55]. Thus the possibility of having similar subclinical systemic inflammation from asymptomatic OA prior to clinical presentation warrants investigation.

### Association in prospective analysis only

Dementia associated with OA only in the prospective analysis. This concurs with a recent systematic review of cross-sectional and case–control studies that reported that people with OA were 20% more likely to have dementia [56]. As dementia is predominantly an ageing problem, the association in the retrospective study may not have been significant because of the low prevalence of dementia in younger adults and difficulty in detecting OA symptoms and fewer consultations for OA in people with dementia. However, the association with SLE could be due to misdiagnosis or miscoding of joint pain symptoms before the actual diagnosis, which needs further investigation. A similar problem may exist for the associations of RA either before or after OA.

This study suggests that although structural changes of OA may appear relatively limited within the skeleton, pathologically and physiologically its effect may possibly be seen in almost every organ. Thus close observation of people with OA through annual assessment in primary care appears warranted, as recommended by the National Institute for Health and Care Excellence (NICE) [57]. Concordantly, the European League Against Rheumatology (EULAR) and NICE have emphasized the importance of diagnosis and management of specific comorbidities and understanding their pattern in OA [57].

### Limitations

There are several caveats to this study. The chances of misclassification of OA because of physician diagnosis rather than full clinical and imaging assessment has been emphasized already. Nevertheless, we tried to optimize identification of symptomatic OA cases through strict inclusion and exclusion criteria using a similar methodology to that of previous studies [58] and there is some reassurance that the codes for hip OA have been shown to have good validity [14]. Misclassification bias for comorbidities is also possible, although most comorbidities in the study have previously been validated [10, 12]. Another important caveat is unavailability of risk factors such as diet and physical activity in the analysis, as these are not routinely recorded within the CPRD. Therefore the estimates in our study may not always relate to direct associations between OA and comorbidities and could have been mediated through other unrecorded factors as mentioned in the discussion. However, the primary aim of the study was to estimate the associations and burden of comorbidities in OA rather than to define risk factors. The associations could to some extent be due to ascertainment bias through increased numbers of hospital or GP visits, especially for the stronger association with rheumatologic conditions. Even though we have not adjusted for the count of hospitalizations, our adjusted estimates were modelled accounting for the number of multimorbidities, which can be considered as a proxy indicator of healthcare visits [59]. Along with the possible Berkesonian bias, a chance of collider bias due to sampling design might exist. However, we matched the controls having a minimum of 36 months of registration and at least one consultation for any reasons. There is also a chance of ascertainment biases due to delayed reporting of OA cases in the database rather than recording the date of first symptom onset. Such bias is inherent to electronic health records, however, our study population age group is quite comparable to that reported by Yu *et al.* [60], showing the consistency in representation of people with OA. We focused more on the possible explanation of the association rather than the plausibility, which is beyond the scope of this study. Our sample size for the prospective analysis was nearly 440 000, with equal numbers of OA cases and matched controls and a maximum follow-up of up to 20 years for 49 comorbidities, making this the first study to provide such a clear picture of the burden of a large number of comorbidities in OA.

In conclusion, the risk of multimorbidity was higher in people with OA. MSK, GI, CVD and psychological conditions were associated with OA both before and after the diagnosis of OA, whereas dementia and SLE were only associated with OA after the diagnosis of OA. The temporal associations reported merit further investigation regarding causality and have important clinical implications with respect to optimal management of OA and its potential comorbidities. Future studies should investigate clustering of the comorbidities and shared risk factors.

## Supplementary data

Supplementary data are available at *Rheumatology* online.

## Supplementary Material

keab067_Supplementary_Data

## References

[1] Feinstein AR. The pre-therapeutic classification of co-morbidity in chronic disease. J Chronic Dis 1970;23:455–68. 26309916 10.1016/0021-9681(70)90054-8

[2] Bähler C, Huber CA, Brüngger B, Reich O. Multimorbidity, health care utilization and costs in an elderly community-dwelling population: a claims data based observational study. BMC Health Serv Res 2015;15:23. 25609174 10.1186/s12913-015-0698-2 PMC4307623

[3] Swain S, Sarmanova A, Coupland C, Doherty M, Zhang W. Comorbidities in osteoarthritis: a systematic review and meta-analysis of observational studies. Arthritis Care Res 2020;72:991–1000. 10.1002/acr.24008 31207113

[4] Parkinson L, Waters DL, Franck L. Systematic review of the impact of osteoarthritis on health outcomes for comorbid disease in older people. Osteoarthritis Cartilage 2017;25:1751–70. 28710026 10.1016/j.joca.2017.07.008

[5] Stubbs B, Aluko Y, Myint PK, Smith TO. Prevalence of depressive symptoms and anxiety in osteoarthritis: a systematic review and meta-analysis. Age Ageing 2016;45:228–35. 26795974 10.1093/ageing/afw001

[6] Wang H, Bai J, He B, Hu X, Liu D. Osteoarthritis and the risk of cardiovascular disease: a meta-analysis of observational studies. Sci Rep 2016;6:39672. 28004796 10.1038/srep39672 PMC5177921

[7] Prados-Torres A, Calderón-Larrañaga A, Hancco-Saavedra J, Poblador-Plou B, van den Akker M. Multimorbidity patterns: a systematic review. J Clin Epidemiol 2014;67:254–66. 24472295 10.1016/j.jclinepi.2013.09.021

[8] van den Akker M, Buntinx F, Knottnerus JA. Comorbidity or multimorbidity: what’s in a name? A review of literature. Eur J Gen Pract 1996;2:65–70.

[9] Ghosh RE, Crellin E, Beatty S et al How Clinical Practice Research Datalink data are used to support pharmacovigilance. Ther Adv Drug Saf 2019;10:2042098619854010. 31210923 10.1177/2042098619854010 PMC6545638

[10] Herrett E, Thomas SL, Schoonen WM, Smeeth L, Hall AJ. Validation and validity of diagnoses in the General Practice Research Database: a systematic review. Br J Clin Pharmacol 2010;69:4–14. 20078607 10.1111/j.1365-2125.2009.03537.x PMC2805870

[11] Clinical Practice Research Datalink. Home page. https://www.cprd.com/.

[12] Khan NF, Harrison SE, Rose PW. Validity of diagnostic coding within the General Practice Research Database: a systematic review. Br J Gen Pract 2010;60:e128–36. 20202356 10.3399/bjgp10X483562 PMC2828861

[13] Jordan KP, Jöud A, Bergknut C et al International comparisons of the consultation prevalence of musculoskeletal conditions using population-based healthcare data from England and Sweden. Ann Rheum Dis 2014;73:212–8. 23345602 10.1136/annrheumdis-2012-202634 PMC3888586

[14] Ferguson RJ, Prieto-Alhambra D, Walker C et al Validation of hip osteoarthritis diagnosis recording in the UK Clinical Practice Research Datalink. Pharmacoepidemiol Drug Saf 2019;28:187–93. 30375101 10.1002/pds.4673

[15] Quality Outcomes Framework (QOF) https://digital.nhs.uk/article/8910/Quality-and-Outcome-Framework-QOF-Indicators-No-Longer-In-QOF-INLIQ-Enhanced-Services-ES-Vaccinations-and-Immunisations-V-I-and-GMS-Core-Contract-CC-extraction-specifications-business-rules-.

[16] Centers for Medicare and Medicaid Services. Chronic conditions. https://www.cms.gov/Research-Statistics-Data-and-Systems/Statistics-Trends-and-Reports/Chronic-Conditions/CC_Main.html.

[17] Charlson ME, Pompei P, Ales KL, MacKenzie CR. A new method of classifying prognostic comorbidity in longitudinal studies: development and validation. J Chronic Dis 1987;40:373–83. 3558716 10.1016/0021-9681(87)90171-8

[18] Sarmanova A, Fernandes GS, Richardson H et al Contribution of central and peripheral risk factors to prevalence, incidence and progression of knee pain: a community-based cohort study. Osteoarthritis Cartilage 2018;26:1461–73. 30099115 10.1016/j.joca.2018.07.013 PMC6215758

[19] Charlson M, Szatrowski TP, Peterson J, Gold J. Validation of a combined comorbidity index. J Clin Epidemiol 1994;47:1245–51. 7722560 10.1016/0895-4356(94)90129-5

[20] Quan H, Li B, Couris CM et al Updating and validating the Charlson comorbidity index and score for risk adjustment in hospital discharge abstracts using data from 6 countries. Am J Epidemiol 2011;173:676–82. 21330339 10.1093/aje/kwq433

[21] Springate DA, Kontopantelis E, Ashcroft DM et al ClinicalCodes: an online clinical codes repository to improve the validity and reproducibility of research using electronic medical records. PLoS One 2014;9:e99825. 24941260 10.1371/journal.pone.0099825 PMC4062485

[22] Payne RA, Mendonca SC, Elliott MN et al Development and validation of the Cambridge Multimorbidity Score. Can Med Assoc J 2020;192:E107–14. 32015079 10.1503/cmaj.190757 PMC7004217

[23] Deyo R. Adapting a clinical comorbidity index for use with ICD-9-CM administrative databases. J Clin Epidemiol 1992;45:613–9. 1607900 10.1016/0895-4356(92)90133-8

[24] National Health Service. What is the body mass index? https://www.nhs.uk/common-health-questions/lifestyle/what-is-the-body-mass-index-bmi/.

[25] Rassen JA, Bartels DB, Schneeweiss S, Patrick AR, Murk W. Measuring prevalence and incidence of chronic conditions in claims and electronic health record databases. Clin Epidemiol 2018;11:1–15. 30588119 10.2147/CLEP.S181242 PMC6301730

[26] Chen G, Lix L, Tu K et al Influence of using different databases and ‘look back’ intervals to define comorbidity profiles for patients with newly diagnosed hypertension: implications for health services researchers. PLoS One 2016;11:e0162074. 27583532 10.1371/journal.pone.0162074 PMC5008755

[27] Greenland S. Multiple comparisons and association selection in general epidemiology. Int J Epidemiol 2008;37:430–4. 18453632 10.1093/ije/dyn064

[28] Benjamini Y, Yekutieli D. The control of the false discovery rate in multiple testing under dependency. Ann Stat 2001;29:1165–88.

[29] Swain S, Sarmanova A, Mallen C et al Trends in incidence and prevalence of osteoarthritis in the United Kingdom: findings from the Clinical Practice Research Datalink (CPRD). Osteoarthritis Cartilage 2020;28:792–801. 32184134 10.1016/j.joca.2020.03.004

[30] Chudasama YV, Khunti KK, Zaccardi F et al Physical activity, multimorbidity, and life expectancy: a UK Biobank longitudinal study. BMC Med 2019;17:108. 31186007 10.1186/s12916-019-1339-0 PMC6560907

[31] Friedman EM, Christ SL, Mroczek DK. Inflammation partially mediates the association of multimorbidity and functional limitations in a national sample of middle-aged and older adults: the MIDUS Study. J Aging Health 2015;27:843–63. 25649677 10.1177/0898264315569453 PMC4499001

[32] Barnes PJ. Mechanisms of development of multimorbidity in the elderly. Eur Respir J 2015;45:790–806. 25614163 10.1183/09031936.00229714

[33] Reeuwijk KG, de Rooij M, van Dijk GM et al Osteoarthritis of the hip or knee: which coexisting disorders are disabling? Clin Rheumatol 2010;29:739–47. 20177725 10.1007/s10067-010-1392-8 PMC2878451

[34] Berenbaum F. Osteoarthritis as an inflammatory disease (osteoarthritis is not osteoarthrosis!). Osteoarthritis Cartilage 2013;21:16–21. 23194896 10.1016/j.joca.2012.11.012

[35] Kirkness CS, Yu J, Asche CV. The effect on comorbidity and pain in patients with osteoarthritis. J Pain Palliat Care Pharmacother 2008;22:336–48. 21923322 10.1080/15360280802536649

[36] Whitehead WE, Palsson OS, Levy RR et al Comorbidity in irritable bowel syndrome. Am J Gastroenterol 2007;102:2767–76. 17900326 10.1111/j.1572-0241.2007.01540.x

[37] Ma CA, Leung YY. Exploring the link between uric acid and osteoarthritis. Front Med (Lausanne) 2017;4:225. 29326934 10.3389/fmed.2017.00225 PMC5733531

[38] Dequeker J, Aerssens J, Luyten FP. Osteoarthritis and osteoporosis: clinical and research evidence of inverse relationship. Aging Clin Exp Res 2003;15:426–39. 14703009 10.1007/BF03327364

[39] Rahman MM, Kopec JA, Cibere J, Goldsmith CH, Anis AH. The relationship between osteoarthritis and cardiovascular disease in a population health survey: a cross-sectional study. BMJ Open 2013;3:e002624. 10.1136/bmjopen-2013-002624 PMC3657665 23674445

[40] Hsu P-S, Lin H-H, Li C-R, Chung W-S. Increased risk of stroke in patients with osteoarthritis: a population-based cohort study. Osteoarthritis Cartilage 2017;25:1026–31. 28300652 10.1016/j.joca.2016.10.027

[41] Findlay DM. Vascular pathology and osteoarthritis. Rheumatology 2007;46:1763–8. 17693442 10.1093/rheumatology/kem191

[42] Louati K, Vidal C, Berenbaum F, Sellam J. Association between diabetes mellitus and osteoarthritis: systematic literature review and meta-analysis. RMD Open 2015;1;1:e000077. 10.1136/rmdopen-2015-000077 PMC4613158 26535137

[43] McGettigan P, Henry D. Cardiovascular risk and inhibition of cyclooxygenase: a systematic review of the observational studies of selective and nonselective inhibitors of cyclooxygenase 2. JAMA 2006;296:1633–44. 16968831 10.1001/jama.296.13.jrv60011

[44] National Institute for Health and Care Excellence. Osteoarthritis: care and management. | Clinical guideline CG177. https://www.nice.org.uk/guidance/cg177/chapter/1-recommendations 31869054

[45] Parmelee PA, Tighe CA, Dautovich ND. Sleep disturbance in osteoarthritis: linkages with pain, disability and depressive symptoms. Arthritis Care Res 2015;67:358–65. 10.1002/acr.22459 PMC4342277 25283955

[46] Krause AJ, Prather AA, Wager TD, Lindquist MA, Walker MP. The pain of sleep loss: a brain characterization in humans. J Neurosci 2019;39:2291–300. 30692228 10.1523/JNEUROSCI.2408-18.2018 PMC6433768

[47] Zak M, Pasiyeshvili L. Chronic gastritis clinical features and stomach functional state during nonsteroidal anti-inflammatory drugs administration in patients with osteoarthritis. Eureka Health Sci 2016;5:17–22.

[48] Arora A, Rajesh S, Bansal K et al Cirrhosis-related musculoskeletal disease: radiological review. Br J Radiol 2016;89:20150450. 27356209 10.1259/bjr.20150450 PMC5124791

[49] Zlateva G, Diazaraque R, Viala-Danten M, Niculescu L. Burden of anemia in patients with osteoarthritis and rheumatoid arthritis in French secondary care. BMC Geriatr 2010;10:2019–6. 10.1186/1471-2318-10-59 PMC2939543 20796267

[50] Kramer SE, Kapteyn TS, Kuik DJ, Deeg DJH. The association of hearing impairment and chronic diseases with psychosocial health status in older age. J Aging Health 2002;14:122–37. 11892756 10.1177/089826430201400107

[51] Goldstein JL, Chan FKL, Lanas A et al Haemoglobin decreases in NSAID users over time: an analysis of two large outcome trials: haemoglobin decreases in NSAID users. Aliment Pharmacol Ther 2011;34:808–16. 21810115 10.1111/j.1365-2036.2011.04790.x PMC3201839

[52] Takatsu M, Higaki M, Kinoshita H, Mizushima Y, Koizuka I. Ear involvement in patients with rheumatoid arthritis. Otol Neurotol 2005;26:755–61. 16015180 10.1097/01.mao.0000178138.19848.bd

[53] Chughtai B, Lee R, Te A, Kaplan S. Role of inflammation in benign prostatic hyperplasia. Rev Urol 2011;13:147–50. 22110398 PMC3221555

[54] Jonas JB, Wei WB, Xu L, Wang YX. Systemic inflammation and eye diseases. The Beijing Eye Study. PLoS One 2018;13:e0204263. 30281641 10.1371/journal.pone.0204263 PMC6169913

[55] Ziegler J. Cancer and arthritis share underlying processes. J Natl Cancer Inst 1998;90:802–3. 9625164 10.1093/jnci/90.11.802

[56] Weber A, Mak SH, Berenbaum F et al Association between osteoarthritis and increased risk of dementia: a systemic review and meta-analysis. Medicine (Baltimore) 2019;98:e14355. 30855434 10.1097/MD.0000000000014355 PMC6417538

[57] Conaghan PG, Dickson J, Grant RL, Guideline Development Group. Care and management of osteoarthritis in adults: summary of NICE guidance. BMJ 2008;336:502–3. 18310005 10.1136/bmj.39490.608009.AD PMC2258394

[58] Swain S, Sarmanova A, Mallen C et al Trends in incidence and prevalence of osteoarthritis in the United Kingdom: findings from the Clinical Practice Research Datalink (CPRD). Osteoarthritis Cartilage 2020;28:792–801. 32184134 10.1016/j.joca.2020.03.004

[59] Cassell A, Edwards D, Harshfield A et al The epidemiology of multimorbidity in primary care: a retrospective cohort study. Br J Gen Pract 2018;68:e245–51. 29530918 10.3399/bjgp18X695465 PMC5863678

[60] Yu D, Jordan KP, Bedson J et al Population trends in the incidence and initial management of osteoarthritis: age-period-cohort analysis of the Clinical Practice Research Datalink, 1992–2013. Rheumatology 2017;56:1902–17. 28977564 10.1093/rheumatology/kex270 PMC5850125

