# Supplementary material for: Temporal relationship between osteoarthritis and comorbidities: a combined case control and cohort study in the UK primary care setting
Source: Rheumatology (Oxford). 2021 Mar 1;60(9):4327–39. doi: 10.1093/rheumatology/keab067 (PMC8410005; doi:10.1093/rheumatology/keab067)
Supplement: keab067_Supplementary_Data [file keab067_supplementary_data.zip › rhe-20-2300-File002.docx]

Supplementary Table S1.1: Code list of key comorbidities

| **Group** | **Diseases** | **Initials of major Read codes** |
| --- | --- | --- |
| Musculoskeletal | Rheumatoid Arthritis | N040.0; N040..; N043… ;N04y… |
|  | Back pain | N11….; N12…; N14….; N3…; Nyu…; S10.…; S49…; S57… |
|  | Gout | C34…; N02z…; N02y…; N022..; N021… |
|  | Osteoporosis | N33… |
|  | Fibromyalgia | N248.00; N239.00; F286… |
|  | Osteoarthritis | N05…; N051… |
| Cardio-vascular | Coronary Heart Disease (Myocardial infraction, Valvular disease, Ischemic heart disease, Angina) | G3…00; G30….; G30z….; G31…; G33…; G34…; G35…; G54….; G57….; Gyu… |
|  | Arterial/Venous (Atherosclerosis/aneurysm/ Embolism) | G70…; G71…; G72….; G74… |
|  | Heart failure (Congestive cardiac failure, heart failure) | G580…; G81….; G232.00; G234.00 |
|  | Hypertension | G20…; G21…; G24…; G25…; G26… |
|  | Peripheral Vascular Disease (Raynaud’s Disease, Buerger’s Disease, Intermittent Claudication) | G73… |
| Respiratory | Asthma | 663…; 66Y… |
|  | COPD | H31…; H32…; H33… |
| Genito-urinary | Chronic Kidney Disease (Stage III) | 1Z12.00; 1Z13.00; 1Z14.00; 1Z15.00; 1Z16.00; K01…; K02… |
|  | Renal stone | 4G4…; 7B07…; K120…. |
|  | Benign Prostatic Hypertrophy (BPH) | K20… |
| Neurological | Stroke (Both Ischemic and Haemorrhagic) | 9Om0.00; 9Om…; 8HBJ.oo; G60…; G61…; F22…; G64…; G63… |
|  | Dementia | E00…; Eu0…; F11…. |
|  | Parkinson’s Disease | F12… |
|  | Migraine | F26…. |
| Psychiatric | Depression | Eu1…; Eu3… |
|  | Psychosis | Eu2Z.11; Eu0z.11 |
|  | Schizophrenia | Eu2… |
| Metabolic/Endocrine | High Cholesterol | C324.00; C322.00; C328.00 |
|  | Diabetes Mellitus | 9OL...00; 2BB…; 2G5…; 66A…; C10…; F42… |
|  | Hyperthyroid | C02… |
|  | Hypothyroidism | C03…; C04… |
| Digestive | Gastritis (Gastric Ulcer, Duodenal Ulcer without haemorrhage, gastritis) | J11…; J12…; J15…14C1… |
|  | Gastrointestinal bleed | J110…; J111….; J120…; J121…; J13….; J14…. J681… |
|  | Gall bladder stone | J65…; 4G2… |
|  | Liver Disease (Cirrhosis and Hepatitis)  Inflammatory Bowel Disease (Crohn’s Disease, Ulcerative colitis) | J61…; J63…; A70… |
|  | Irritable Bowel Syndrome | J521… |
| Other | Hearing Impairment | F59…. |
|  | Sleep Disorder (Insomnia and Apnoea) | Fy0… |
|  | Anaemia (Any type) | D00… |
|  | Vision Problem (Non-cataract) | F49… |
|  | Cataract | F46… |

The codes are initials of the Read codes representing the comorbidities. COPD- Chronic obstructive pulmonary disease

Supplementary Table S1.2: Comorbidities in the time periods before the index date in OA cases (any joint) and matched controls

|  | **0-5 years** | | | | **0-10 years** | | | | **0-15 years** | | | | **0-20 years** | | | |
| --- | --- | --- | --- | --- | --- | --- | --- | --- | --- | --- | --- | --- | --- | --- | --- | --- |
|  | **Non-OA(N=220547)** | | **OA(N=221128)** | | **Non-OA (219234)** | | **OA (219496)** | | **Non-OA (215778)** | | **OA(N=216157)** | | **Non-OA(N=208799)** | | **OA(N=209601)** | |
|  | n | % | n | % | n | % | n | % | n | % | n | % | n | % | n | % |
| **Musculoskeletal** |  |  |  |  |  |  |  |  |  |  |  |  |  |  |  |  |
| Ankylosing Spondylitis | 674 | 0.30 | 1092 | 0.49 | 1358 | 0.61 | 2163 | 0.98 | 1889 | 0.87 | 2891 | 1.33 | 2158 | 1.03 | 3258 | 1.55 |
| Back pain | 21452 | 9.73 | 30343 | 13.72 | 40443 | 18.44 | 55334 | 25.20 | 54194 | 25.11 | 73749 | 34.11 | 61835 | 29.61 | 84092 | 40.12 |
| Gout | 2162 | 0.98 | 3625 | 1.64 | 3564 | 1.62 | 5995 | 2.73 | 4382 | 2.03 | 7325 | 3.38 | 4829 | 2.31 | 8013 | 3.82 |
| Osteoporosis | 2622 | 1.19 | 3680 | 1.66 | 4028 | 1.84 | 5267 | 2.39 | 4664 | 2.61 | 5961 | 2.75 | 4896 | 2.34 | 6260 | 2.98 |
| Polymyalgia | 659 | 0.29 | 1285 | 0.58 | 980 | 0.45 | 1885 | 0.86 | 1161 | 0.54 | 2129 | 0.98 | 1243 | 0.59 | 2226 | 1.06 |
| Rheumatoid Arthritis | 387 | 0.17 | 1015 | 0.46 | 710 | 0.32 | 1586 | 0.72 | 865 | 0.40 | 1904 | 0.88 | 972 | 0.46 | 1956 | 0.93 |
| Sjogren’s syndrome | 71 | 0.03 | 171 | 0.08 | 137 | 0.06 | 265 | 0.12 | 173 | 0.08 | 314 | 0.14 | 202 | 0.09 | 340 | 0.16 |
| Systemic lupus erythematosus | 26 | 0.01 | 46 | 0.02 | 49 | 0.02 | 80 | 0.03 | 73 | 0.03 | 109 | 0.05 | 81 | 0.04 | 122 | 0.05 |
| Fibromyalgia | 490 | 0.22 | 1192 | 0.54 | 827 | 0.37 | 1829 | 0.83 | 1027 | 0.47 | 2129 | 0.98 | 1073 | 0.51 | 2162 | 1.03 |
| Fatigue | 915 | 0.41 | 1363 | 0.62 | 1445 | 0.66 | 2099 | 0.95 | 1693 | 0.78 | 2386 | 1.10 | 1739 | 0.83 | 2453 | 1.17 |
| **Respiratory** |  |  |  |  |  |  |  |  |  |  |  |  |  |  |  |  |
| Asthma | 3636 | 1.64 | 5338 | 2.41 | 7458 | 3.40 | 10628 | 4.84 | 10482 | 4.86 | 14770 | 6.83 | 12320 | 5.90 | 17029 | 8.12 |
| COPD | 2984 | 1.35 | 4209 | 1.90 | 5886 | 2.68 | 8126 | 3.70 | 8043 | 3.72 | 11088 | 5.12 | 9296 | 4.45 | 12642 | 6.05 |
| **Genito-Urinary** |  |  |  |  |  |  |  |  |  |  |  |  |  |  |  |  |
| Chronic kidney disease | 5549 | 2.51 | 6789 | 3.07 | 7369 | 3.36 | 8768 | 3.99 | 7513 | 3.48 | 8960 | 4.14 | 7527 | 3.60 | 8965 | 4.27 |
| Benign prostatic hypertrophy^ | 2899 | 1.31 | 4038 | 1.83 | 4884 | 2.22 | 6543 | 2.98 | 5972 | 2.76 | 7901 | 3.65 | 6365 | 3.05 | 8436 | 4.02 |
| Renal stone | 542 | 0.24 | 698 | 0.31 | 989 | 0.45 | 1261 | 0.57 | 1325 | 0.61 | 1635 | 0.76 | 1567 | 0.75 | 1923 | 0.91 |
| **Neuro/Psychiatric** |  |  |  |  |  |  |  |  |  |  |  |  |  |  |  |  |
| Stroke | 6025 | 2.73 | 7141 | 3.22 | 10314 | 4.70 | 11902 | 5.42 | 13042 | 6.04 | 14826 | 6.85 | 14200 | 6.80 | 16158 | 7.70 |
| Dementia | 741 | 0.33 | 908 | 0.41 | 929 | 0.42 | 1036 | 0.47 | 978 | 0.45 | 1061 | 0.49 | 990 | 0.47 | 1068 | 0.51 |
| Epilepsy | 414 | 0.19 | 511 | 0.23 | 702 | 0.30 | 897 | 0.41 | 966 | 0.45 | 1196 | 0.55 | 1125 | 0.54 | 1376 | 0.65 |
| Multiple sclerosis | 127 | 0.57 | 124 | 0.56 | 236 | 0.11 | 230 | 0.10 | 329 | 0.15 | 293 | 0.13 | 433 | 0.20 | 348 | 0.17 |
| Parkinson’s Disease | 318 | 0.14 | 481 | 0.22 | 450 | 0.20 | 629 | 0.29 | 489 | 0.23 | 680 | 0.31 | 502 | 0.24 | 696 | 0.33 |
| Migraine | 2487 | 1.12 | 3561 | 1.61 | 5093 | 2.32 | 7065 | 3.21 | 7203 | 3.33 | 9802 | 4.53 | 8489 | 4.06 | 11359 | 5.41 |
| Depression | 9051 | 4.10 | 13588 | 6.14 | 17610 | 8.03 | 25398 | 11.57 | 24076 | 11.15 | 34044 | 15.74 | 27362 | 13.10 | 38417 | 18.32 |
| Psychosis | 154 | 0.07 | 161 | 0.07 | 291 | 0.13 | 268 | 0.12 | 377 | 0.17 | 354 | 0.16 | 419 | 0.20 | 398 | 0.19 |
| Schizophrenia | 325 | 0.14 | 389 | 0.17 | 657 | 0.29 | 692 | 0.31 | 888 | 0.41 | 928 | 0.43 | 1034 | 0.49 | 1073 | 0.51 |
| **Cancer** | 3697 | 1.67 | 4287 | 1.94 | 5951 | 2.71 | 6795 | 3.09 | 7294 | 3.38 | 8248 | 3.81 | 7984 | 3.80 | 8972 | 4.28 |
| **Circulatory** |  |  |  |  |  |  |  |  |  |  |  |  |  |  |  |  |
| Coronary heart disease | 5059 | 2.29 | 6390 | 2.89 | 9472 | 4.32 | 12171 | 5.54 | 12496 | 5.79 | 16162 | 7.47 | 14262 | 6.83 | 18302 | 8.73 |
| Arterial/Venous | 513 | 0.23 | 731 | 0.33 | 825 | 0.37 | 1123 | 0.51 | 989 | 0.46 | 1337 | 0.62 | 1062 | 0.51 | 1429 | 0.68 |
| Heart failure | 1045 | 0.47 | 1795 | 0.81 | 1568 | 0.71 | 2658 | 1.21 | 1777 | 0.82 | 3011 | 1.39 | 1847 | 0.88 | 3113 | 1.48 |
| Hypertension | 18204 | 8.25 | 20969 | 9.48 | 32449 | 14.80 | 37418 | 17.04 | 41348 | 19.16 | 48042 | 22.22 | 46012 | 22.03 | 53659 | 25.60 |
| Peripheral vascular disease | 1767 | 0.80 | 2734 | 1.23 | 2939 | 1.34 | 4411 | 2.00 | 3614 | 1.67 | 5191 | 2.40 | 3906 | 1.87 | 5539 | 2.64 |
| **Metabolic** |  |  |  |  |  |  |  |  |  |  |  |  |  |  |  |  |
| High Cholesterol | 9875 | 4.47 | 12467 | 5.63 | 16604 | 7.57 | 20458 | 9.32 | 20291 | 9.40 | 24714 | 11.43 | 21865 | 10.47 | 26558 | 12.67 |
| Diabetes Mellitus | 6188 | 2.80 | 7954 | 3.59 | 9945 | 4.53 | 12677 | 5.77 | 11856 | 5.49 | 15129 | 6.99 | 12656 | 6.06 | 16147 | 7.70 |
| Hyperthyroid | 665 | 0.30 | 712 | 0.32 | 1205 | 0.55 | 1294 | 0.59 | 1579 | 0.73 | 1732 | 0.80 | 1843 | 0.88 | 2047 | 0.97 |
| Hypothyroidism | 4075 | 1.84 | 5067 | 2.29 | 7050 | 3.21 | 8732 | 3.97 | 8922 | 4.13 | 11096 | 5.13 | 9793 | 4.69 | 12276 | 5.85 |
| **Digestive** |  |  |  |  |  |  |  |  |  |  |  |  |  |  |  |  |
| Gastritis | 2771 | 1.25 | 4069 | 1.84 | 4915 | 2.24 | 7070 | 3.22 | 6542 | 3.03 | 9198 | 4.25 | 7551 | 3.61 | 10527 | 5.02 |
| Gastrointestinal bleed | 672 | 0.30 | 1032 | 0.47 | 1133 | 0.52 | 1675 | 0.76 | 1431 | 0.66 | 2072 | 0.96 | 1570 | 0.75 | 2253 | 1.07 |
| Gall bladder stone | 2490 | 1.13 | 3438 | 1.55 | 4296 | 1.95 | 6077 | 2.76 | 5602 | 2.59 | 7971 | 3.68 | 6461 | 3.09 | 9189 | 4.38 |
| Inflammatory Bowel Disease | 2548 | 1.15 | 3695 | 1.67 | 4514 | 2.05 | 6379 | 2.90 | 5824 | 2.69 | 7983 | 3.69 | 6409 | 3.06 | 8704 | 4.15 |
| Liver Disease | 329 | 0.15 | 508 | 0.23 | 506 | 0.23 | 796 | 0.36 | 637 | 0.29 | 950 | 0.44 | 689 | 0.32 | 1029 | 0.49 |
| Irritable bowel syndrome | 3134 | 1.42 | 4787 | 2.16 | 6266 | 2.85 | 9261 | 4.21 | 8653 | 4.01 | 12589 | 5.82 | 10015 | 4.79 | 14335 | 6.83 |
| **Others** |  |  |  |  |  |  |  |  |  |  |  |  |  |  |  |  |
| Hearing | 7193 | 3.26 | 9172 | 4.14 | 11807 | 5.38 | 14748 | 6.71 | 14329 | 6.64 | 17855 | 8.26 | 15587 | 7.46 | 19315 | 9.21 |
| Vision problem | 510 | 0.23 | 625 | 0.28 | 860 | 0.39 | 1015 | 0.46 | 1059 | 0.49 | 1209 | 0.56 | 1136 | 0.54 | 1313 | 0.62 |
| Psoriasis | 1286 | 0.58 | 1751 | 0.79 | 2379 | 1.08 | 3127 | 1.42 | 3174 | 1.47 | 4086 | 1.89 | 3655 | 1.75 | 4602 | 2.19 |
| Scleroderma | 17 | 0.01 | 29 | 0.01 | 37 | 0.02 | 41 | 0.02 | 46 | 0.02 | 51 | 0.02 | 54 | 0.02 | 55 | 0.02 |
| Sleep Disorder | 2061 | 0.93 | 2877 | 1.30 | 3169 | 1.44 | 4340 | 1.97 | 3677 | 1.70 | 4978 | 2.30 | 3820 | 1.82 | 5148 | 2.45 |
| Tuberculosis | 112 | 0.05 | 139 | 0.06 | 215 | 0.09 | 269 | 0.12 | 310 | 0.14 | 360 | 0.17 | 342 | 0.16 | 417 | 0.19 |
| Anaemia | 2389 | 1.08 | 3385 | 1.53 | 4010 | 1.83 | 5268 | 2.40 | 4927 | 2.28 | 6269 | 2.90 | 5406 | 2.59 | 6732 | 3.21 |
| Cataract | 5643 | 2.56 | 6897 | 3.11 | 8581 | 3.91 | 10346 | 4.71 | 9791 | 4.53 | 11715 | 5.42 | 10218 | 4.89 | 12179 | 5.81 |
| **Comorbidities (count)** |  |  |  |  |  |  |  |  |  |  |  |  |  |  |  |  |
| No comorbidity | 131897 | 59.33 | 109920 | 49.44 | 95710 | 43.05 | 73856 | 33.22 | 81303 | 36.57 | 61335 | 27.59 | 77845 | 35.01 | 59752 | 26.88 |
| Single comorbidity | 57054 | 25.66 | 64354 | 28.95 | 60358 | 27.15 | 59574 | 26.80 | 56157 | 25.26 | 51005 | 22.94 | 51546 | 23.18 | 44541 | 20.03 |
| Any two comorbidities | 22617 | 10.17 | 30264 | 13.61 | 35762 | 16.08 | 42647 | 19.18 | 39244 | 17.65 | 43511 | 19.57 | 38897 | 17.49 | 41327 | 18.59 |
| Any three comorbidities | 7618 | 3.42 | 11901 | 5.35 | 17810 | 8.01 | 24647 | 11.09 | 23302 | 10.48 | 30336 | 13.64 | 25282 | 11.37 | 31429 | 14.14 |
| Four or more | 3104 | 1.39 | 5851 | 2.63 | 12650 | 5.69 | 21568 | 9.70 | 22284 | 10.02 | 36103 | 16.24 | 28720 | 12.92 | 45241 | 20.35 |

COPD- Chronic Obstructive Pulmonary Disease; ^only for men

Supplementary Table S1.3: Association between any OA (any joint) and comorbidities in time periods prior to the index date (Expanded version)

|  | **0-20 years** | | **0-15 years** | | **0-10 years** | | **0-5 years** | |
| --- | --- | --- | --- | --- | --- | --- | --- | --- |
|  | **Unadjusted OR** | **Adjusted OR^#^** | **Unadjusted OR** | **Adjusted OR^#^** | **Unadjusted OR** | **Adjusted OR^#^** | **Unadjusted OR** | **Adjusted OR^#^** |
| **>=2 comorbidities** | 1.86 (1.83-1.88)* | 1.71(1.69-1.74)* | 1.80(1.77-1.82)* | 1.66(1.63-1.68)* | 1.71(1.68-1.73)* | 1.58(1.56-1.60)* | 1.63(1.60-1.65)* | 1.53(1.49-1.55)* |
| **Musculoskeletal** |  |  |  |  |  |  |  |  |
| Ankylosing Spondylitis | 1.53 (1.45-1.62)* | 1.53 (1.44-1.62)* | 1.55 (1.46-1.64) | 1.56 (1.46-1.65)* | 1.61 (1.50-1.72)* | 1.63 (1.52-1.75)* | 1.61 (1.46-1.77)* | 1.63 (1.47-1.79)* |
| Back pain | 1.70 (1.67-1.72)* | 1.67 (1.64-1.69)* | 1.61 (1.59-1.64)* | 1.59 (1.56-1.61)* | 1.52 (1.50-1.55)* | 1.51 (1.48-1.53)* | 1.47 (1.44-1.50)* | 1.45 (1.43-1.48)* |
| Gout | 1.69 (1.64-1.76)* | 1.52 (1.46-1.57)* | 1.70 (1.63-1.77)* | 1.52 (1.45-1.58)* | 1.69 (1.63-1.77)* | 1.52 (1.45-1.59)* | 1.67 (1.58-1.76)* | 1.49 (1.41-1.58)* |
| Osteoporosis | 1.27 (1.22-1.32)* | 1.41 (1.35-1.47)* | 1.27 (1.22-1.32)* | 1.41 (1.35-1.46)* | 1.29 (1.23-1.34)* | 1.42 (1.36-1.49)* | 1.36 (1.29-1.43)* | 1.49 (1.42-1.58)* |
| Polymyalgia | 1.80 (1.68-1.93)* | 1.74 (1.62-1.87)* | 1.84 (1.71-1.98)* | 1.78 (1.65-1.92)* | 1.92 (1.78-2.08)* | 1.86 (1.72-2.01)* | 1.93 (1.76-2.12)* | 1.86 (1.69-2.05)* |
| Rheumatoid Arthritis | 1.97 (1.83-2.13)* | 1.95 (1.80-2.11)* | 2.16 (1.99-2.34)* | 2.14 (1.97-2.32)* | 2.18 (1.99-2.39)* | 2.17 (1.98-2.38)* | 2.51 (2.23-2.83)* | 2.50 (2.21-2.82)* |
| Sjogren’s syndrome | 1.64 (1.38-1.96)* | 1.67 (1.39-2.00)* | 1.77 (1.47-2.13)* | 1.82 (1.50-2.20)* | 1.86 (1.52-2.29)* | 1.94 (1.56-2.40)* | 2.31 (1.75-3.05)* | 2.47 (1.85-3.30)* |
| Systemic lupus erythematosus | 1.49 (1.12-1.98) | 1.54 (1.15-2.07) | 1.48 (1.09-1.99) | 1.54 (1.10-2.05) | 1.63 (1.14-2.33) | 1.59 (1.09-2.29) | 1.77 (1.09-2.86) | 1.72 (1.05-2.82) |
| Fibromyalgia | 1.95 (1.81-2.10)* | 1.89 (1.75-2.04)* | 2.01 (1.86-2.16)* | 1.95 (1.80-2.11)* | 2.12 (1.95-2.31)* | 2.07 (1.89-2.25)* | 2.27 (2.03-2.53)* | 2.19 (1.96-2.45)* |
| Fatigue | 1.42 (1.33-1.51)* | 1.42 (1.32-1.51)* | 1.42 (1.33-1.51)* | 1.42 (1.33-1.52)* | 1.46 (1.36-1.56)* | 1.46 (1.36-1.57)* | 1.49 (1.36-1.62)* | 1.48 (1.36-1.62)* |
| **Respiratory** |  |  |  |  |  |  |  |  |
| Asthma | 1.41 (1.38-1.45)* | 1.33 (1.30-1.37)* | 1.44 (1.40-1.48)* | 1.35 (1.31-1.39)* | 1.44 (1.40-1.49)* | 1.35 (1.31-1.39)* | 1.46 (1.40-1.53)* | 1.37 (1.31-1.43)* |
| COPD | 1.40 (1.37-1.45)* | 1.35 (1.31-1.39)* | 1.42 (1.38-1.46)* | 1.36 (1.32-1.41)* | 1.40 (1.36-1.46)* | 1.36 (1.31-1.41)* | 1.40 (1.34-1.47)* | 1.37 (1.30-1.43)* |
| **Genito-Urinary** |  |  |  |  |  |  |  |  |
| Chronic kidney disease | 1.25 (1.20-1.29)* | 1.12 (1.08-1.16)* | 1.24 (1.20-1.29)* | 1.12 (1.08-1.16)* | 1.24 (1.20-1.29)* | 1.12(1.08-1.16)* | 1.27 (1.22-1.32)* | 1.15 (1.10-1.19)* |
| Benign prostatic hypertrophy^ | 1.38 (1.32-1.43)* | 1.38 (1.33-1.43)* | 1.37 (1.32-1.42)* | 1.37 (1.32-1.42)* | 1.37 (1.31-1.42)* | 1.37 (1.32-1.43)* | 1.39 (1.32-1.46)* | 1.37 (1.32-1.46)* |
| Renal stone | 1.22 (1.14-1.31) | 1.16 (1.09-1.25)* | 1.23 (1.15-1.33)* | 1.16 (1.08-1.26)* | 1.28 (1.17-1.39)* | 1.21 (1.11-1.32)* | 1.28 (1.14-1.43)* | 1.21 (1.08-1.36)* |
| **Neuro/Psychiatric** |  |  |  |  |  |  |  |  |
| Stroke | 1.17 (1.14-1.20)* | 1.15 (1.11-1.19)* | 1.17 (1.14-1.20)* | 1.15 (1.11-1.19)* | 1.18 (1.15-1.21)* | 1.15 (1.12-1.19)* | 1.19 (1.15-1.24)* | 1.17 (1.13-1.22)* |
| Dementia | 1.07 (0.97-1.17) | 1.09 (0.99-1.19) | 1.07 (0.98-1.17) | 1.09 (1.00-1.20) | 1.10 (1.01-1.21) | 1.13 (1.03-1.24) | 1.21 (1.09-1.33)* | 1.23 (1.11-1.36)* |
| Epilepsy | 1.20 (1.11-1.30)* | 1.18 (1.08-1.29)* | 1.22 (1.12-1.33)* | 1.20 (1.10-1.31)* | 1.26 (1.14-1.39)* | 1.24 (1.11-1.37)* | 1.20 (1.05-1.37) | 1.17 (1.03-1.35) |
| Multiple sclerosis | 0.79 (0.68-0.91)* | 0.80 (0.69-0.93)* | 0.88 (0.75-1.03) | 0.89 (0.75-1.04) | 0.97 (0.81-1.16) | 0.95 (0.78-1.14) | 0.98 (0.76-1.25) | 0.95 (0.72-1.20) |
| Parkinson’s Disease | 1.36 (1.21-1.53)* | 1.39 (1.23-1.57)* | 1.36 (1.21-1.53)* | 1.39 (1.24-1.57)* | 1.36 (1.21-1.54)* | 1.39 (1.22-1.57)* | 1.46 (1.27-1.68)* | 1.47 (1.27-1.70)* |
| Migraine | 1.36 (1.32-1.39)* | 1.37 (1.33-1.41)* | 1.38 (1.33-1.42)* | 1.39 (1.34-1.43)* | 1.40 (1.35-1.45)* | 1.42 (1.36-1.47)* | 1.42 (1.34-1.49)* | 1.44 (1.37-1.53)* |
| Depression | 1.53 (1.50-1.56)* | 1.49 (1.46-1.52)* | 1.52 (1.49-1.55)* | 1.49 (1.46-1.52)* | 1.52 (1.49-1.55)* | 1.49 (1.46-1.52)* | 1.52 (1.48-1.56)* | 1.49 (1.45-1.54)* |
| Psychosis | 0.94 (0.82-1.08) | 0.86 (0.75-1.00) | 0.93 (0.81-1.08) | 0.86 (0.74-0.99) | 0.90 (0.76-1.07) | 0.83 (0.69-0.98) | 1.02 (0.82-1.27) | 0.95 (0.75-1.19) |
| Schizophrenia | 1.03 (0.95-1.12) | 0.95 (0.87-1.04) | 1.03 (0.95-1.14) | 0.95 (0.86-1.05) | 1.05 (0.94-1.17) | 0.97 (0.87-1.08) | 1.17 (1.01-1.36) | 1.08 (0.92-1.26) |
| **Cancer** | 1.13 (1.09-1.17)* | 1.12 (1.09-1.16)* | 1.14 (1.09-1.17)* | 1.12 (1.09-1.16)* | 1.15 (1.10-1.18)* | 1.12 (1.08-1.17)* | 1.15 (1.10-1.20)* | 1.12 (1.08-1.18)* |
| **Circulatory** |  |  |  |  |  |  |  |  |
| Coronary heart disease | 1.33 (1.30-1.36)* | 1.24 (1.21-1.27)* | 1.34 (1.31-1.37)* | 1.24 (1.20-1.27)* | 1.31 (1.28-1.35)* | 1.22 (1.18-1.25)* | 1.26 (1.21-1.32)* | 1.17 (1.12-1.21)* |
| Arterial/Venous | 1.34 (1.23-1.45)* | 1.29 (1.19-1.41)* | 1.34 (1.24-1.46)* | 1.30 (1.19-1.42)* | 1.34 (1.23-1.47)* | 1.30 (1.19-1.43)* | 1.39 (1.24-1.57)* | 1.35 (1.20-1.52)* |
| Heart failure | 1.72 (1.62-1.82)* | 1.52 (1.43-1.62)* | 1.73 (1.62-1.83)* | 1.52 (1.44-1.62)* | 1.72 (1.61-1.84)* | 1.52 (1.43-1.63)* | 1.72 (1.59-1.86)* | 1.53 (1.41-1.65)* |
| Hypertension | 1.24 (1.22-1.26)* | 1.08 (1.06-1.10)* | 1.22 (1.20-1.24)* | 1.08 (1.05-1.09)* | 1.18 (1.16-1.20)* | 1.06 (1.04-1.07)* | 1.15 (1.12-1.17)* | 1.04 (1.02-1.06)* |
| Peripheral vascular disease | 1.41 (1.35-1.47)* | 1.45 (1.39-1.51)* | 1.42 (1.36-1.49)* | 1.45 (1.39-1.53)* | 1.48 (1.41-1.55)* | 1.51 (1.44-1.59)* | 1.49 (1.41-1.59)* | 1.54 (1.45-1.64)* |
| **Metabolic/Endocrine** |  |  |  |  |  |  |  |  |
| High Cholesterol | 1.27 (1.24-1.29)* | 1.18 (1.16-1.20)* | 1.26 (1.24-1.29)* | 1.18 (1.15-1.20)* | 1.27 (1.24-1.29)* | 1.18 (1.15-1.21)* | 1.27 (1.23-1.31)* | 1.20 (1.16-1.23)* |
| Diabetes Mellitus | 1.31 (1.27-1.34)* | 1.06 (1.03-1.09)* | 1.30 (1.27-1.34)* | 1.06 (1.03-1.08)* | 1.29 (1.26-1.33)* | 1.06 (1.02-1.10)* | 1.29 (1.24-1.33)* | 1.06 (1.02-1.09)* |
| Hyperthyroid | 1.10 (1.03-1.17)* | 1.09 (1.02-1.16)* | 1.09 (1.01-1.17) | 1.08 (1.00-1.15) | 1.06 (0.98-1.15) | 1.05 (0.97-1.14) | 1.05 (0.94- 1.16) | 1.04 (0.93-1.15) |
| Hypothyroidism | 1.27 (1.23-1.30)* | 1.18 (1.15-1.22)* | 1.25 (1.22-1.29)* | 1.18 (1.14-1.21)* | 1.24 (1.20-1.28)* | 1.17 (1.12-1.20)* | 1.22 (1.17-1.28)* | 1.16 (1.11-1.21)* |
| **Digestive** |  |  |  |  |  |  |  |  |
| Gastritis | 1.42 (1.37-1.46)* | 1.42 (1.36-1.45)* | 1.43 (1.37-1.47)* | 1.42 (1.37-1.47)* | 1.45 (1.39-1.50)* | 1.45 (1.39-1.50)* | 1.46 (1.39-1.53)* | 1.45 (1.38-1.52)* |
| Gastrointestinal bleed | 1.43 (1.34-1.53)* | 1.42 (1.33-1.52)* | 1.45 (1.36-1.55)* | 1.43 (1.34-1.54)* | 1.47 (1.36-1.59)* | 1.44 (1.33-1.56)* | 1.52 (1.38-1.68)* | 1.49 (1.34-1.64)* |
| Gall bladder stone | 1.44 (1.39-1.49)* | 1.27 (1.22-1.31)* | 1.43 (1.36-1.49)* | 1.27 (1.22-1.31)* | 1.42 (1.37-1.48)* | 1.26 (1.21-1.31)* | 1.37 (1.30-1.44)* | 1.23 (1.17-1.30)* |
| Inflammatory bowel disease | 1.38 (1.33-1.43)* | 1.36 (1.32-1.41)* | 1.39 (1.34-1.44)* | 1.38 (1.33-1.43)* | 1.43 (1.37-1.48)* | 1.42 (1.36-1.47)* | 1.45 (1.38-1.53)* | 1.44 (1.36-1.52)* |
| Liver Disease | 1.47 (1.33-1.62)* | 1.42 (1.29-1.57)* | 1.46 (1.32-1.62)* | 1.42 (1.27-1.56)* | 1.55 (1.38-1.73)* | 1.48 (1.32-1.67)* | 1.49 (1.30-1.72)* | 1.45 (1.26-1.68)* |
| Irritable bowel syndrome | 1.47 (1.43-1.51)* | 1.52(1.47-1.56)* | 1.50(1.45-1.54)* | 1.54(1.49-1.58)* | 1.50(1.46-1.55)* | 1.55(1.49-1.60)* | 1.54(1.47-1.61)* | 1.58(1.51-1.66)* |
| **Others** |  |  |  |  |  |  |  |  |
| HIV infection/AIDS | 1.99 (0.75-5.32) | 2.08 (0.76-5.75) | 1.66 (0.65-4.22) | 1.64 (0.62-4.33) | 1.38 (0.52-3.62) | 1.49 (0.54-4.16) | 2.99 (0.81-11.08) | 3.17 (0.84-12.03) |
| Hearing | 1.26 (1.24-1.29)* | 1.26 (1.23-1.29)* | 1.27 (1.24-1.30)* | 1.26 (1.23-1.29)* | 1.26 (1.23-1.29)* | 1.26 (1.22-1.29)* | 1.27 (1.23-1.31)* | 1.26 (1.22-1.30)* |
| Psoriasis | 1.24 (1.19-1.30)* | 1.20 (1.14-1.25)* | 1.27 (1.22-1.33)* | 1.22 (1.16-1.28)* | 1.30 (1.23-1.37)* | 1.24 (1.17-1.31)* | 1.32 (1.23-1.42)* | 1.26 (1.17-1.36)* |
| Scleroderma | 0.98 (0.67-1.43) | 0.97 (0.65-1.44) | 1.06 (0.71-1.59) | 1.05 (0.69-1.59) | 1.05 (0.67-1.65) | 1.02 (0.64-1.64) | 1.59 (0.86-2.91) | 1.76 (0.94-3.30) |
| Sleep Disorder | 1.43 (1.36-1.49)* | 1.35 (1.28-1.41)* | 1.44 (1.37-1.51)* | 1.35 (1.29-1.42)* | 1.45 (1.38-1.53)* | 1.37 (1.30-1.44)* | 1.45 (1.37-1.55)* | 1.37 (1.28-1.46)* |
| Tuberculosis | 1.21 (1.04-1.39) | 1.25 (1.08-1.45) | 1.16 (0.99-1.35) | 1.19 (1.02-1.39) | 1.25 (1.04-1.50) | 1.24 (1.04-1.50) | 1.21 (0.94-1.55) | 1.23 (0.95-1.59) |
| Anaemia | 1.25 (1.20-1.29)* | 1.25 (1.21-1.30)* | 1.28(1.23-1.33)* | 1.28(1.23-1.33)* | 1.32(1.26-1.37)* | 1.31(1.26-1.37)* | 1.41(1.33-1.49)* | 1.40(1.32-1.48)* |
| Vision problem | 1.15 (1.07-1.25) | 1.11 (1.02-1.21) | 1.14 (1.05-1.24) | 1.11 (1.00-1.19) | 1.17 (1.07-1.29) | 1.13 (1.03-1.24) | 1.21 (1.07-1.36) | 1.17 (1.03-1.32) |
| Cataract | 1.23(1.19-1.27)* | 1.21(1.17-1.24)* | 1.23(1.20-1.27)* | 1.21(1.17-1.25)* | 1.24(1.20-1.28)* | 1.21(1.17-1.25)* | 1.23(1.19-1.28)* | 1.21(1.16-1.25)* |

*p <0.01 adjusted for multiple testing using ‘False discovery rate’; ^#^Adjusted for age, gender, BMI, Smoking, Alcohol use, multimorbidity and index year ^Only for men

COPD- Chronic Obstructive Pulmonary Disease

Supplementary Table S1.4: Association between joint specific OA and comorbidities prior to the index date

|  | Hip | | Knee | | Wrist/Hand | | Ankle/Foot | |
| --- | --- | --- | --- | --- | --- | --- | --- | --- |
|  | 0-1 year | 0-20 years | 0-1 year | 0-20 years | 0-1 year | 0-20 years | 0-1 year | 0-20 years |
|  | Adjusted OR  (95% CI) | Adjusted OR  (95% CI) | Adjusted OR  (95% CI) | Adjusted OR  (95% CI) | Adjusted OR  (95% CI) | Adjusted OR  (95% CI) | Adjusted OR  (95% CI) | Adjusted OR  (95% CI) |
| **Musculoskeletal** |  |  |  |  |  |  |  |  |
| Ankylosing Spondylitis | 1.37(0.76-2.49) | 1.62(1.39-1.90)* | 0.91(0.57-1.43) | 1.55(1.37-1.73)* | 2.01(0.90-4.46) | 1.57(1.24-1.96)* | 3.58(0.57-22.34) | 1.40(0.96-2.00) |
| Back pain | 2.22(1.99-2.47)* | 1.66(1.59-1.73)* | 1.21(1.11-1.31)* | 1.51(1.47-1.56)* | 1.28(1.06-1.54) | 1.58(1.49-1.69)* | 1.25(0.98-1.60) | 1.59(1.45-1.73)* |
| Gout | 0.74(0.50-1.09) | 1.21(1.09-1.35)* | 1.03(0.81-1.31) | 1.49(1.39-1.61)* | 0.86(0.45-1.59) | 1.70(1.39-2.08)* | 3.07(1.61-5.84) | 2.56(2.01-3.14)* |
| Osteoporosis | 1.62(1.23-2.14) | 1.30(1.16-1.46)* | 1.25(1.00-1.56) | 1.25(1.13-1.34)* | 0.88(0.56-1.38) | 1.26(1.05-1.53) | 1.69(0.83-3.42) | 1.34(1.04-1.85) |
| Polymyalgia | 2.16(1.24-3.74) | 1.39(1.14-1.69)* | 1.20(0.79-1.83) | 1.56(1.32-1.77)* | 1.65(0.53-5.13) | 1.58(1.07-2.35) | 0.38(0.07-1.91) | 1.38(0.81-2.37) |
| Rheumatoid Arthritis | 2.95(1.29-6.72) | 1.25(0.99-1.63) | 1.21(0.75-1.93) | 1.43(1.21-1.70)* | 4.76(1.54-14.79) | 1.57(0.99-1.99) | 1.98(0.47-8.30) | 1.30(0.62-1.72) |
| Sjogren’s syndrome | 0.80(0.05-13.88) | 1.93(1.08-3.47) | 1.59(0.49-5.09) | 1.47(1.04-2.09) | - | 1.32(0.63-2.74) | - | 1.30(0.32-5.22) |
| SLE | - | - | 1.92(0.10-36.03) | 1.19(0.62-2.29) | - | 0.38(0.09-1.38) | - | - |
| Fibromyalgia | 1.12(0.52-2.45) | 1.51(1.17-1.92) | 1.87(1.21-2.87) | 1.75(1.49-2.05)* | 0.97(0.35-2.74) | 1.53(1.14-2.07) | 1.46(0.49-4.31) | 1.29(0.81-2.03) |
| Fatigue | 1.54(0.93-2.55) | 1.32(1.09-1.60) | 1.21(0.85-1.71) | 1.38(1.21-1.59)* | 2.32(1.05-5.12) | 1.42(1.09-1.84) | 1.41(0.58-3.40) | 1.10(0.66-1.53) |
| **Respiratory** |  |  |  |  |  |  |  |  |
| Asthma | 1.16(0.87-1.55) | 1.19(1.11-1.28)* | 1.45(1.17-1.79) | 1.38(1.31-1.46)* | 1.46(0.90-2.37) | 1.31(1.18-1.47)* | 1.01(0.54-1.88) | 1.38(1.18-1.62)* |
| COPD | 1.42(1.06-1.90) | 1.20(1.11-1.31)* | 1.56(1.25-1.94)* | 1.33(1.25-1.41)* | 0.73(0.41-1.28) | 1.23(1.07-1.41) | 0.71(0.32-1.57) | 1.25(1.02-1.52) |
| **Genito-Urinary** |  |  |  |  |  |  |  |  |
| Chronic kidney disease | 0.97(0.80-1.18) | 1.10(0.99-1.21) | 1.02(0.88-1.18) | 1.04(0.97-1.13) | 0.80(0.54-1.19) | 0.92(0.77-1.10) | 1.08(0.68-1.71) | 1.05(0.82-1.34) |
| Benign prostatic hypertrophy^ | 1.67(1.26-2.21)* | 1.40(1.27-1.55)* | 1.09(0.90-1.33) | 1.32(1.25-1.43)* | 1.80(1.06-3.07) | 1.56(1.29-1.89)* | 1.54(0.88-2.71) | 1.40(1.10-1.72)* |
| Renal stone | 0.99(0.47-2.12) | 1.05(0.87-1.28) | 2.30(1.30-4.08) | 1.41(1.15-1.51)* | 1.47(0.32-6.78) | 1.22(0.86-1.73) | 0.49(0.12-2.04) | 1.60(1.07-2.39) |
| **Neuro/Psychiatric** |  |  |  |  |  |  |  |  |
| Stroke | 1.09(0.89-1.34) | 1.09(1.00-1.16) | 1.20(1.03-1.39) | 1.20(1.10-1.22)* | 1.40(0.98-1.99) | 1.24(1.09-1.40)* | 1.37(0.83-2.24) | 1.17(0.90-1.28) |
| Dementia | 1.44(0.89-2.34) | 1.11(0.86-1.44) | 1.28(0.89-1.82) | 0.93(0.77-1.12) | 0.63(0.23-1.70) | 0.72(0.44-1.17) | 1.89(0.47-7.53) | 0.96(0.45-2.01) |
| Epilepsy | 1.95(0.76-4.97) | 1.27(0.99-1.61) | 1.21(0.67-2.18) | 1.29(1.09-1.51) | 1.89(0.41-8.73) | 1.12(0.76-1.66) | 0.96(0.06-15.51) | 0.81(0.45-1.44) |
| Multiple sclerosis | 1.09(0.29-4.21) | 0.73(0.48-1.10) | 0.72(0.19-2.79) | 0.96(0.70-1.29) | 1.71(0.10-29.60) | 0.67(0.34-1.32) | 2.09(0.33-13.28) | 0.63(0.25-1.61) |
| Parkinson’s Disease | 1.34(0.66-2.73) | 0.87(0.62-1.23) | 1.43(0.84-2.45) | 1.20(0.96-1.52) | 0.15(0.02-1.17) | 1.11(0.55-2.24) | - | 1.49(0.66-3.35) |
| Migraine | 1.05(0.73-1.53) | 1.16(1.06-1.28)* | 1.29(0.99-1.68) | 1.35(1.27-1.44)* | 1.35(0.83-2.18) | 1.47(1.30-1.67)* | 1.23(0.56-2.69) | 1.38(1.14-1.67)* |
| Depression | 1.43(1.18-1.73)* | 1.32(1.25-1.39)* | 1.52(1.34-1.73)* | 1.46(1.43-1.49)* | 1.26(0.97-1.64) | 1.48(1.34-1.57)* | 1.50(1.05-2.15) | 1.40(1.27-1.60)* |
| Psychosis | - | 1.09(0.70-1.71) | 2.59(0.94-7.16) | 1.05(0.82-1.44) | - | 0.58(0.30-1.12) |  |  |
| Schizophrenia | 0.90(0.31-2.58) | 0.99(0.77-1.29) | 1.51(0.69-3.27) | 1.15(0.96-1.38) | 1.98(0.18-21.97) | 0.83(0.55-1.25) | - | 0.56(0.38-1.13) |
| **Cancer** | 1.13(0.86-1.47) | 1.24(1.13-1.35)* | 0.84(0.68-1.01) | 1.11(1.03-1.17) | 0.92(0.56-1.51) | 0.92(0.79-1.07) | 1.25(0.68-2.31) | 1.16(0.93-1.45) |
| **Circulatory** |  |  |  |  |  |  |  |  |
| Coronary Heart Disease | 1.06(0.83-1.36) | 1.18(1.10-1.26)* | 1.09(0.92-1.29) | 1.15(1.09-1.21)* | 0.94(0.58-1.53) | 1.02(0.90-1.16) | 1.07(0.59-1.93) | 1.38(1.17-1.63)* |
| Arterial/Venous | 1.00(0.55-1.85) | 1.34(1.09-1.65) | 1.87(1.13-3.10) | 1.21(1.03-1.42) | 1.88(0.51-6.93) | 0.96(0.63-1.52) | 0.56(0.09-3.19) | 0.84(0.47-1.51) |
| Heart failure | 1.72(1.11-2.67) | 1.38(1.16-1.63)* | 1.27(0.93-1.74) | 1.34(1.20-1.53)* | 1.44(0.44-4.69) | 1.17(0.89-1.83) | 1.13(0.33-3.90) | 1.57(1.03-2.38) |
| Hypertension | 1.10(0.97-1.24) | 1.12(1.07-1.17)* | 1.06(0.97-1.16) | 1.10(1.07-1.15)* | 0.85(0.67-1.07) | 1.02(0.95-1.10) | 0.93(0.69-1.24) | 1.08(0.97-1.20) |
| Peripheral vascular disease | 1.86(1.34-2.59)* | 1.37(1.21-1.55)* | 1.58(1.22-2.04)* | 1.29(1.16-1.38)* | 1.89(1.04-3.43) | 1.50(1.22-1.86)* | 1.60(0.82-3.11) | 1.44(1.09-1.89) |
| **Metabolic/Endocrine** |  |  |  |  |  |  |  |  |
| High Cholesterol | 1.18(0.99-1.39) | 1.15(1.09-1.22)* | 1.13(1.01-1.26) | 1.14(1.09-1.19)* | 1.29(0.99-1.67) | 1.22(1.11-1.35)* | 1.16(0.81-1.66) | 1.11(1.01-1.33) |
| Diabetes Mellitus | 1.06(0.86-1.31) | 1.06(0.98-1.13) | 1.04(0.90-1.19) | 1.02(0.98-1.08) | 1.11(0.78-1.57) | 0.97(0.85-1.10) | 1.23(0.78-1.96) | 0.95(0.79-1.13) |
| Hyperthyroid | 0.93(0.45-1.92) | 1.13(0.94-1.38) | 0.96(0.57-1.64) | 1.13(0.99-1.30) | 0.38(0.10-1.43) | 1.05(0.79-1.39) | 0.56(0.14-2.25) | 1.15(0.72-1.85) |
| Hypothyroidism | 1.34(1.02-1.74) | 1.23(1.13-1.34)* | 1.04(0.94-1.39) | 1.17(1.10-1.24)* | 1.13(0.75-1.75) | 1.21(1.07-1.38)* | 1.21(0.65-2.24) | 1.11(0.91-1.37) |
| **Digestive** |  |  |  |  |  |  |  |  |
| Gastritis | 1.21(0.90-1.61) | 1.22(1.11-1.34)* | 1.53(1.23-1.89)* | 1.39(1.30-1.47)* | 1.30(0.79-2.14) | 1.26(1.09-1.45)* | 0.76(0.33-1.74) | 1.45(1.18-1.78)* |
| Gastrointestinal bleed | 2.62(1.44-4.79) | 1.49(1.23-1.80)* | 1.86(1.27-2.72) | 1.37(1.21-1.56)* | 1.06(0.42-2.68) | 1.35(0.99-1.83) | 0.92(0.19-4.31) | 1.48(0.96-2.31) |
| Gall bladder stone | 0.95(0.67-1.34) | 1.22(1.11-1.35)* | 1.11(0.87-1.42) | 1.33(1.25-1.43)* | 1.08(0.65-1.77) | 1.31(1.13-1.52)* | 0.31(0.11-0.85) | 1.45(1.14-1.83)* |
| Inflammatory Bowel Disease | 1.14(0.84-1.54) | 1.23(1.11-1.37)* | 1.25(0.99-1.56) | 1.35(1.26-1.44)* | 1.32(0.82-2.12) | 1.22(1.04-1.40) | 0.92(0.42-2.05) | 1.63(1.29-2.06)* |
| Liver Disease | 0.91(0.37-2.25) | 1.14(0.85-1.55) | 1.33(0.75-2.33) | 1.32(1.08-1.62) | - | 1.18(0.71-1.96) | - | 1.51(0.74-3.06) |
| Irritable Bowel Syndrome | 1.17(0.89-1.57) | 1.33(1.22-1.46)* | 1.28(0.99-1.56) | 1.38(1.29-1.48)* | 1.30(0.80-2.10) | 1.25(1.08-1.42) | 0.98(0.47-2.02) | 1.61(1.24-2.02)* |
| **Others** |  |  |  |  |  |  |  |  |
| HIV infection/AIDS | - | 0.86(0.13-5.86) | - | 2.05(0.20-20.69) | - | - | - | - |
| Hearing | 1.19(0.98-1.45) | 1.14(1.06-1.21)* | 1.32(1.16-1.50)* | 1.24(1.18-1.29)* | 1.26(0.94-1.69) | 1.31(1.18-1.46)* | 1.07(0.72-1.60) | 1.41(1.23-1.67)* |
| Psoriasis | 1.04(0.66-1.63) | 1.07(0.93-1.22) | 1.10(.80-1.53) | 1.13(1.04-1.25) | 2.31(1.02-5.25) | 1.07(0.86-1.29) | 1.92(0.61-6.04) | 1.15(1.01-1.81) |
| Scleroderma | - | 1.29(0.33-5.06) | 1.15(0.08-16.35) | 072(0.26-1.52) | - | - |  |  |
| Sleep Disorder | 1.15(0.81-1.62) | 1.25(1.-8-1.43) | 1.29(1.02-1.65) | 1.44(1.26-1.53)* | 1.74(1.05-2.86) | 1.44(1.15-1.78)* | 1.87(0.85-4.10) | 1.48(1.09-2.02) |
| Tuberculosis | 0.92(0.7-12.02) | 0.86(0.56-1.32) | 0.90(0.16-5.04) | 1.35(1.00-1.84) | - | 3.44(1.23-9.58) | - | 2.56(0.93-7.07) |
| Anaemia | 1.35(0.96-1.90) | 1.21(1.08-1.36)* | 1.29(1.04-1.59) | 1.26(1.16-1.35)* | 1.19(0.76-1.89) | 1.31(1.10-1.53) | 0.83(0.39-1.79) | 1.14(0.87-1.49) |
| Vision problem | 0.88(0.43-1.80) | 1.05(0.83-1.33) | 0.87(0.51-1.48) | 1.11(0.95-1.31) | 1.21(0.32-4.58) | 1.27(0.85-1.90) | - | 0.77(0.39-1.51) |
| Cataract | 0.84(0.45-1.24) | 1.02(0.81-1.31) | 0.79(0.45-1.42) | 1.15(0.99-1.29) | 1.25(0.38-4.72) | 1.25(0.83-1.86) | - | 0.74(0.33-1.48) |

**P-*value <0.05 adjusted for multiple testing using ‘False discovery rate’; Adjusted for age, gender, body mass index (BMI), Smoking, Alcohol use, multimorbidity and index year; ^for men only; SLE – Systemic Lupus Erythematous; COPD- Chronic Obstructive Pulmonary Disease;

Blank space indicates OR could not be calculated due to insufficient numbers.

Joint specific analysis for each comorbidity is given in Supplementary table S1.4. For hip joint OA, 20 years before the index date leading comorbidities having a positive association with hip OA were back pain (aOR 1.66; 95% CI 1.59-1.73), ankylosing spondylitis (aOR 1.62; 95% CI 1.39-1.90), fibromyalgia (aOR 1.51; 95% CI 1.17-1.92), gastro-intestinal bleeding (aOR 1.49; 95% CI 1.23-1.80), polymyalgia (aOR 1.39; 95% CI 1.14-1.69) and depression (aOR 1.32; 95% CI 1.25-1.39). Leading comorbidities associated with knee OA before 20 years of index date were musculoskeletal conditions such as fibromyalgia (aOR 1.75; 95% CI 1.49-2.05), polymyalgia (aOR 1.56; 95% CI 1.32-1.77), ankylosing spondylitis (aOR 1.55; 95% CI 1.37-1.73), back pain (aOR 1.51; 95% CI 1.47-1.56), gout (aOR 1.49; 95% CI 1.39-1.61) and depression (aOR 1.46; 95% CI 1.43-1.49) and sleep disorder (aOR 1.44; 95% CI 1.26-1.53).

For wrist and hand OA, leading associations were seen with gout (aOR 1.70; 95% CI 1.39-2.08), back pain (aOR 1.58; 95% CI 1.49-1.69), ankylosing spondylitis (aOR 1.57; 95% CI 1.24-1.96), benign prostate hypertrophy (aOR 1.56; 95% CI 1.29-1.89), hypertension (aOR 1.50; 95% CI 1.22-1.86), depression (aOR 1.48; 95% CI 1.34-1.57) and migraine (aOR 1.47; 95% CI 1.30-1.67). Comorbidities associated with ankle/foot OA before 20 years of index date were gout (aOR 2.56; 95% CI 2.01-3.14), inflammatory bowel disease (aOR 1.63; 95% CI 1.29-2.06), back pain (aOR 1.59; 95% CI 1.45-1.23), gastritis (aOR 1.45; 95% CI 1.18-1.78), gall bladder stone(aOR 1.45; 95% CI 1.14-1.83), hearing problems (aOR 1.41; 95% CI 1.23-1.67) and benign prostate hypertrophy (aOR 1.40; 95% CI 1.10-1.22).

Supplementary Table S1.5: Cumulative probabilities (%) of incident comorbidities after index date in people with OA at index date and matched controls

|  |  | **Osteoarthritis (cases)** | | | | | **Non-Osteoarthritis (controls)** | | |
| --- | --- | --- | --- | --- | --- | --- | --- | --- | --- |
|  | **5 years** | **10 years** | **15 years** | **20 years** |  | **5 years** | **10 years** | **15 years** | **20 years** |
| **Multimorbidity** | 27.35 | 53.00 | 68.37 | 77.40 |  | 19.51 | 42.91 | 59.56 | 70.73 |
| **Musculoskeletal** |  |  |  |  |  |  |  |  |  |
| Ankylosing Spondylitis | 0.43 | 0.81 | 1.14 | 1.41 |  | 0.25 | 0.50 | 0.72 | 0.98 |
| Back pain | 20.52 | 34.30 | 44.18 | 50.89 |  | 14.06 | 25.35 | 34.30 | 41.12 |
| Gout | 2.12 | 4.42 | 6.73 | 8.63 |  | 1.27 | 2.67 | 4.24 | 5.91 |
| Osteoporosis | 2.41 | 4.98 | 7.86 | 10.52 |  | 1.84 | 4.24 | 7.15 | 10.15 |
| Polymyalgia | 0.71 | 1.39 | 2.07 | 2.94 |  | 0.42 | 0.94 | 1.52 | 2.42 |
| Rheumatoid Arthritis | 0.78 | 1.40 | 1.96 | 2.51 |  | 0.18 | 0.37 | 0.54 | 0.73 |
| Sjogren’s syndrome | 0.09 | 0.16 | 0.26 | 0.26 |  | 0.04 | 0.09 | 0.12 | 0.23 |
| Systemic lupus erythematous | 0.03 | 0.06 | 0.08 | 0.10 |  | 0.01 | 0.03 | 0.04 | 0.05 |
| Fibromyalgia | 0.74 | 1.24 | 1.66 | 2.03 |  | 0.20 | 0.38 | 0.53 | 0.69 |
| Fatigue | 0.78 | 1.53 | 2.27 | 3.00 |  | 0.53 | 1.97 | 1.57 | 2.08 |
| **Respiratory** |  |  |  |  |  |  |  |  |  |
| Asthma | 1.89 | 3.37 | 4.61 | 5.94 |  | 1.45 | 2.53 | 3.43 | 4.29 |
| COPD | 1.99 | 4.05 | 6.16 | 8.64 |  | 1.52 | 3.33 | 5.34 | 7.07 |
| **Genito-Urinary** |  |  |  |  |  |  |  |  |  |
| Chronic kidney disease | 6.33 | 14.87 | 20.27 | 24.43 |  | 5.31 | 12.80 | 17.89 | 22.40 |
| Benign prostatic hypertrophy^ | 2.09 | 3.92 | 5.56 | 7.18 |  | 1.59 | 3.12 | 4.54 | 5.75 |
| Renal stone | 0.36 | 0.76 | 1.12 | 1.59 |  | 0.28 | 0.59 | 0.96 | 1.34 |
| **Neuro/Psychiatric** |  |  |  |  |  |  |  |  |  |
| Stroke | 4.05 | 8.29 | 12.75 | 16.98 |  | 3.25 | 6.92 | 10.79 | 14.93 |
| Dementia | 1.50 | 3.94 | 7.30 | 11.00 |  | 0.98 | 2.85 | 5.71 | 9.22 |
| Epilepsy | 0.25 | 0.50 | 0.75 | 1.17 |  | 0.18 | 0.38 | 0.57 | 0.70 |
| Multiple sclerosis | 0.05 | 0.10 | 0.12 | 0.16 |  | 0.05 | 0.08 | 0.11 | 0.12 |
| Parkinson’s Disease | 0.39 | 0.81 | 1.19 | 1.58 |  | 0.24 | 0.55 | 0.94 | 1.50 |
| Migraine | 1.36 | 2.37 | 3.19 | 4.11 |  | 1.00 | 1.78 | 2.38 | 2.85 |
| Depression | 6.95 | 11.70 | 15.73 | 19.43 |  | 4.29 | 7.64 | 10.50 | 13.33 |
| Psychosis | 0.09 | 0.18 | 0.28 | 0.38 |  | 0.09 | 0.17 | 0.26 | 0.35 |
| Schizophrenia | 0.19 | 0.36 | 0.51 | 0.66 |  | 0.16 | 0.30 | 0.43 | 0.53 |
| **Cancer** | 4.37 | 9.54 | 14.81 | 20.07 |  | 2.73 | 6.37 | 10.48 | 14.90 |
| **Circulatory** |  |  |  |  |  |  |  |  |  |
| Coronary heart disease | 3.23 | 6.12 | 8.70 | 11.29 |  | 2.37 | 4.52 | 6.68 | 8.48 |
| Arterial/Venous | 0.54 | 1.15 | 1.89 | 2.55 |  | 0.34 | 0.84 | 1.38 | 1.95 |
| Heart failure | 1.47 | 2.89 | 4.43 | 5.92 |  | 0.73 | 1.64 | 2.73 | 3.91 |
| Hypertension | 11.85 | 21.01 | 27.99 | 33.75 |  | 10.24 | 18.90 | 25.59 | 31.54 |
| Peripheral vascular disease | 1.47 | 2.88 | 4.26 | 5.52 |  | 0.98 | 2.00 | 3.07 | 3.91 |
| **Metabolic/Endocrine** |  |  |  |  |  |  |  |  |  |
| High Cholesterol | 7.00 | 12.59 | 16.95 | 19.36 |  | 5.76 | 10.82 | 14.71 | 17.77 |
| Diabetes Mellitus | 5.28 | 11.18 | 17.30 | 23.19 |  | 3.94 | 8.43 | 13.58 | 18.98 |
| Hyperthyroid | 0.37 | 0.71 | 1.00 | 1.29 |  | 0.31 | 0.58 | 0.84 | 1.10 |
| Hypothyroidism | 2.41 | 4.46 | 6.26 | 7.45 |  | 2.02 | 3.92 | 5.53 | 6.79 |
| **Digestive** |  |  |  |  |  |  |  |  |  |
| Gastritis | 2.45 | 4.77 | 7.01 | 9.23 |  | 1.41 | 3.00 | 4.60 | 6.24 |
| Gastrointestinal bleed | 0.70 | 1.42 | 2.09 | 2.65 |  | 0.40 | 0.83 | 1.35 | 1.94 |
| Gall bladder stone | 1.96 | 3.91 | 5.96 | 7.66 |  | 1.33 | 2.72 | 4.10 | 5.49 |
| Inflammatory bowel disease | 1.97 | 3.87 | 5.44 | 6.85 |  | 1.31 | 2.58 | 3.84 | 4.60 |
| Liver Diseases | 0.31 | 0.64 | 1.05 | 1.40 |  | 0.17 | 0.39 | 0.57 | 0.79 |
| Irritable bowel syndrome | 2.00 | 3.48 | 4.63 | 5.66 |  | 1.33 | 2.31 | 3.13 | 3.85 |
| **Others** |  |  |  |  |  |  |  |  |  |
| HIV infection/AIDS | 0.00 | 0.0001 | 0.0001 | 0.0001 |  | 0.00 | 0.00 | 0.00 | 0.00 |
| Hearing | 5.73 | 11.74 | 17.82 | 24.18 |  | 4.74 | 10.17 | 16.06 | 21.59 |
| Psoriasis | 0.70 | 1..28 | 1.81 | 2.28 |  | 0.57 | 1.05 | 1.47 | 1.77 |
| Scleroderma | 0.02 | 0.04 | 0.06 | 0.11 |  | 0.02 | 0.03 | 0.05 | 0.06 |
| Sleep Disorder | 1.59 | 3.06 | 4.38 | 5.58 |  | 1.11 | 2.04 | 2.95 | 3.78 |
| Tuberculosis | 0.06 | 0.12 | 0.17 | 0.25 |  | 0.05 | 0.09 | 0.11 | 0.14 |
| Anaemia | 2.70 | 5.45 | 8.22 | 11.04 |  | 1.56 | 3.53 | 5.65 | 7.53 |
| Vision problem | 0.37 | 0.79 | 1.15 | 1.57 |  | 0.28 | 0.68 | 1.11 | 1.53 |
| Cataract | 4.74 | 9.87 | 15.30 | 20.45 |  | 4.18 | 9.10 | 14.35 | 19.20 |

COPD- Chronic Obstructive Pulmonary Disease

^ only for men
