# Supplementary material for: Temporal relationship between osteoarthritis and comorbidities: a combined case control and cohort study in the UK primary care setting
Source: Rheumatology (Oxford). 2021 Mar 1;60(9):4327–39. doi: 10.1093/rheumatology/keab067 (PMC8410005; doi:10.1093/rheumatology/keab067)
Supplement: keab067_Supplementary_Data [file keab067_supplementary_data.zip › rhe-20-2300-File003.docx]

**Sensitivity analysis**

Of 221,807 incident OA cases 22,333 (10.1%) were without any of the comorbidities of interest on the index date. An equal number of controls (22,333) without comorbidities were matched to these cases for age (+2 years), gender, practice area and index year. The mean age was 56.7 years (SD- 13.6) in OA cases and 56.5 years (SD- 13.6) in matched non-OA controls, 52.4% in both groups being women. Details of the distribution of covariates are shown in Supplementary Table S2.1.

Those with incident OA were significantly more likely to develop 25 specific comorbidities than non-OA controls (Supplementary Table S2.2). The cumulative probability of developing multimorbidity was higher in OA compared to controls. The cumulative probabilities of having multimorbidity at 5, 15 and 20 years following the index date were 0.64%, 22.14% and 52.93% in people with incident OA and 0.25%, 15.53% and 38.00% in controls, respectively (Supplementary Figure S2.1). A comparison of the adjusted OR and HR for comorbidities before and after OA diagnosis is depicted in Supplementary Figure S2.2. Twenty-five conditions had significant bidirectional associations with OA both over 20 years before and after the index date, specifically MSK (RA, fibromyalgia, ankylosing spondylitis), GI [GI bleeding, liver disease, gall bladder stone, IBS, inflammatory bowel disease (IBD)], CVD (heart failure, coronary heart disease, high cholesterol, stroke) and psychological (depression). Dementia was significant in the prospective analysis only, and infectious diseases tended to have no association with OA. However, 15 comorbidities including hypertension, chronic obstructive pulmonary disease (COPD), thyroid, migraine, Parkinson’s disease, and asthma were significantly associated with OA retrospectively only. Comorbidities with the strongest associations were fibromyalgia (aHR 5.29; 95% CI 2.65-10.50), rheumatoid arthritis (aHR 4.31; 95% CI 2.68-6.92), liver disease (aHR 3.36; 95% CI 1.89-5.97), sleep problems (aHR 1.95; 95% CI 1.50-2.55), gastrointestinal (GI) bleeding (aHR 1.93; 95% CI 1.14-3.27), ankylosing spondylitis (aHR 1.85; 95% CI 1.13-3.10), dementia (aHR 1.77; 95% CI 1.32-2.38), heart failure (aHR 1.62; 95% CI 1.10-2.39), osteoporosis (aHR 1.61; 95% CI 1.32-1.98) and anaemia (aHR 1.57; 95% CI 1.19-2.09). (Supplementary Table S2.2)

A comparison of the HR of two different samples are given in supplementary figure S2.3 shows majority overlapping with the association.

Supplementary Table S2.1: Characteristics of incident OA patients and matched controls at index date (without any comorbidities at the index date)

|  | Incident OA  (n=22,333) | Controls  (n=22,333) | Unadjusted  Odds Ratio (95%CI) |
| --- | --- | --- | --- |
| **Age (years)** |  |  |  |
| <40 years | 2484(12.09) | 2577(12.57) | NA |
| 40-49 years | 4017(17.99) | 4101(18.36) | NA |
| 50-59 years | 6594(29.53) | 6496(29.09) | NA |
| 60-69 years | 5477(24.52) | 5490(24.58) | NA |
| 70-79 years | 2949(13.20) | 2885(12.92) | NA |
| 80-89 years | 776(3.47) | 746(3.34) | NA |
| >90 years | 36(0.16) | 38(0.17) | NA |
| **Gender** |  |  |  |
| Men | 10622(47.56) | 10622(17.56) | NA |
| Women | 11711(52.43) | 11711(52.43) | NA |
| **BMI (kg/m^2^)** |  |  |  |
| <18.5 | 279(1.25) | 452(2.02) | 0.82(0.71-0.96)* |
| 18.5- 24.9 | 6214(27.85) | 8493(38.04) | Reference |
| 25.0-29.9 | 8314(37.26) | 8367(37.48) | 1.38(1.32-1.44)* |
| >30 | 7503(33.63) | 5010(22.44) | 2.09(1.99-2.20)* |
| **Alcohol consumption (units/week)** |  |  |  |
| Never | 4318(19.33) | 4139(18.53) | Reference |
| Ex-drinker | 536(2.40) | 465(2.08) | 1.10(0.96-1.26) |
| Current 1-9 | 8052(36.05) | 8245(36.92) | 0.93(0.88-0.99)* |
| Current >=10 | 4147(18.57) | 4237(18.97) | 0.93(0.87-0.98)* |
| Current Unknown | 5277(23.63) | 5246(23.49) | 0.96(0.91-1.02) |
| **Smoking Status** |  |  |  |
| Never smoked | 11715(52.45) | 12160(54.44) | Reference |
| Ex-smoker | 6101(27.31) | 5774(25.85) | 1.10(1.05-1.12)* |
| Current smoker | 4516(20.22) | 4399(19.69) | 1.06(1.02-1.11)* |
| Age in years total  (Mean, SD) | 56.71(13.55) | 56.53(13.58) |  |
| Age in years Men  (Mean, SD) | 55.71(13.31) | 55.53(13.34) |  |
| Age in years Women  (Mean, SD) | 57.62(13.70) | 57.44(13.73) |  |
| BMI in Kg/M^2^  (Mean, SD) | 28.44(5.68) | 26.80(5.05) |  |

#Adjusted by age, index year and first year of registration

*p <0.05

NA-not applicable; BMI- Body mass index; SD- Standard deviation

Supplementary Table S2.2: Hazard ratio and 95% confidence interval for each comorbidity comparing incident OA cases and controls without any comorbidities at the index date

|  | **Unadjusted HR**  **(95% CI)** | **Adjusted HR**  **(95% CI)** | **p value** |
| --- | --- | --- | --- |
| **Two or more comorbidities** | 1.38(1.31-1.45) | 1.34(1.28-1.41) | 0.001* |
| **Musculoskeletal** |  |  |  |
| Ankylosing Spondylitis | 1.86(1.13-3.05) | 1.85(1.13-3.10) | 0.028* |
| Back pain | 1.46(1.37-1.55) | 1.45(1.36-1.54) | 0.001* |
| Gout | 1.57(1.30-1.91) | 1.40(1.15-1.70) | 0.002* |
| Osteoporosis | 1.38(1.13-1.69) | 1.61(1.32-1.98) | 0.001* |
| Polymyalgia | 1.48(0.92-2.38) | 1.60(0.99-2.59) | 0.088 |
| Rheumatoid Arthritis | 4.25(2.65-6.82) | 4.31(2.68-6.92) | 0.001* |
| Sjogren’s syndrome | 2.12(0.62-7.26) | 2.22(0.64-7.70) | 0.279 |
| Systemic lupus erythematosus | 2.24(0.41-12.30) | 2.45(0.44-13.62) | 0.369 |
| Fibromyalgia | 5.28(2.66-10.48) | 5.29(2.65-10.50) | 0.001* |
| Fatigue | 1.25(0.89-1.76) | 1.25(0.89-1.77) | 0.265 |
| **Respiratory** |  |  |  |
| Asthma | 1.15(0.97-1.36) | 1.09(0.92-1.29) | 0.368 |
| COPD | 1.22(0.99-1.49) | 1.19(0.98-1.46) | 0.088 |
| **Genito-Urinary** |  |  |  |
| Chronic Kidney Disease | 1.17(1.02-1.35) | 1.14(0.99-1.32) | 0.098 |
| Benign prostatic hypertrophy^ | 1.55(1.27-1.88) | 1.56(1.28-1.90) | 0.001* |
| Renal stone | 0.91(0.61-1.37) | 0.81(0.54-1.22) | 0.369 |
| **Neuro/Psychiatric** |  |  |  |
| Stroke | 1.15(1.05-1.22) | 1.14(1.06-1.24) | 0.001* |
| Dementia | 1.43(1.07-1.90) | 1.77(1.32-2.38) | 0.001* |
| Epilepsy | 0.87(0.49-1.54) | 0.88(0.49-1.56) | 0.698 |
| Multiple sclerosis | 0.85(0.32-2.28) | 0.75(0.28-2.03) | 0.608 |
| Parkinson’s disease | 1.21(0.68-2.12) | 1.32(0.74-2.34) | 0.398 |
| Migraine | 1.28(1.02-1.59) | 1.27(1.02-1.59) | 0.064 |
| Depression | 1.58(1.43-1.74) | 1.55(1.40-1.71) | 0.001* |
| Psychosis | 1.44(0.63-3.35) | 1.38(0.59-3.24) | 0.488 |
| Schizophrenia | 1.30(0.73-2.31) | 1.25(0.70-2.23) | 0.488 |
| **Cancer** | 1.46(1.27-1.69) | 1.43(1.24-1.65) | 0.001* |
| **Circulatory** |  |  |  |
| Coronary Heart Disease | 1.27(1.07-1.51) | 1.19(1.01-.142) | 0.075 |
| Arterial/Venous | 1.18(0.72-1.96) | 1.27(0.76-2.11) | 0.398 |
| Heart failure | 1.61(1.09-2.35) | 1.62(1.10-2.39) | 0.022* |
| Hypertension | 1.15(1.06-1.24) | 1.06(0.98-1.14) | 0.225 |
| Peripheral Vascular Disease | 1.56(1.18-2.05) | 1.57(1.19-2.09) | 0.002* |
| **Metabolic/Endocrine** |  |  |  |
| High Cholesterol | 1.19(1.08-1.33) | 1.15(1.04-1.29) | 0.014* |
| Diabetes Mellitus | 1.43(1.26-1.62) | 1.26(1.11-1.43) | 0.001* |
| Hyperthyroid | 1.06(0.68-1.66) | 1.05(0.67-1.66) | 0.832 |
| Hypothyroidism | 1.21(1.02-1.45) | 1.15(0.96-1.37) | 0.204 |
| **Digestive** |  |  |  |
| Gastritis | 1.46(1.18-1.79) | 1.41(1.15-1.74) | 0.002* |
| Gastrointestinal bleed | 1.95(1.16-3.28) | 1.93(1.14-3.27) | 0.027* |
| Gall bladder stone | 1.48(1.18-1.85) | 1.31(1.05-1.64) | 0.034* |
| Inflammatory bowel disease | 1.33(1.08-1.65) | 1.31(1.06-1.62) | 0.026* |
| Liver Disease | 3.55(2.01-6.26) | 3.36(1.89-5.97) | 0.001* |
| Irritable bowel syndrome | 1.43(1.26-1.62) | 1.43(1.27-1.63) | 0.001* |
| **Others** |  |  |  |
| HIV infection/AIDS | 1.23(0.08-19.65) | 0.85(0.50-14.21) | 0.907 |
| Hearing | 1.30(1.15-1.46) | 1.31(1.16-1.48) | 0.001* |
| Psoriasis | 1.37(1.05-1.79) | 1.31(1.00-1.72) | 0.082 |
| Scleroderma | 3.57(0.37-34.52) | 3.79(0.39-37.19) | 0.324 |
| Sleep Disorder | 1.95(1.50-2.53) | 1.95(1.50-2.55) | 0.001* |
| Tuberculosis | 0.47(0.16-1.33) | 0.48(0.17-1.38) | 0.251 |
| Anaemia | 1.58(1.28-1.93) | 1.57(1.27-1.95) | 0.001* |
| Vision problem | 1.35(0.64-2.85) | 1.56(0.73-3.34) | 0.324 |
| Cataract | 1.07(0.99-1.5) | 1.12(1.04-1.21) | 0.005* |

Adjusted for age, sex, BMI, alcohol use, smoking and index date

***p <0.05 ‘False discovery rate’ (FDR) adjusted; p-y person years; COPD- Chronic Obstructive Pulmonary Disease

Supplementary Figure S2.1: Cumulative probabilities of developing multimorbidity in cases with OA and matched non-OA controls without any comorbidities at the index date


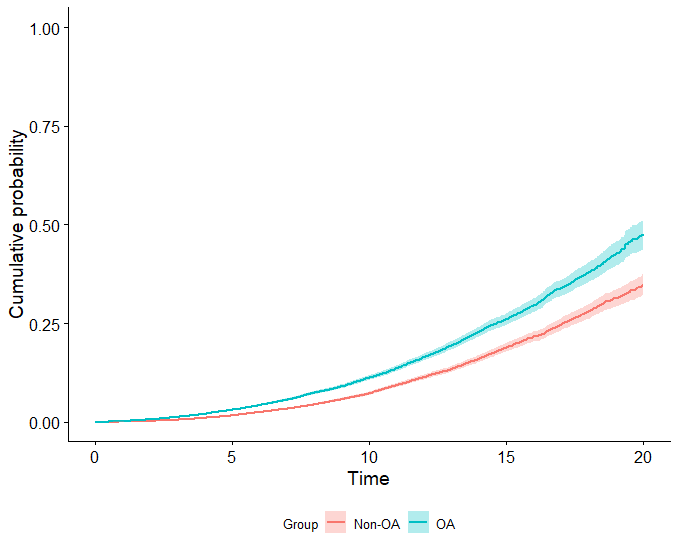


OA: Osteoarthritis (cases); Non-OA: Non-Osteoarthritis (controls)

Supplementary Figure S2.2: Comparison of adjusted Odds Ratio and Hazard Ratio for comorbidities in OA for 20 years observation period among OA and matched controls without any comorbidities at the index date


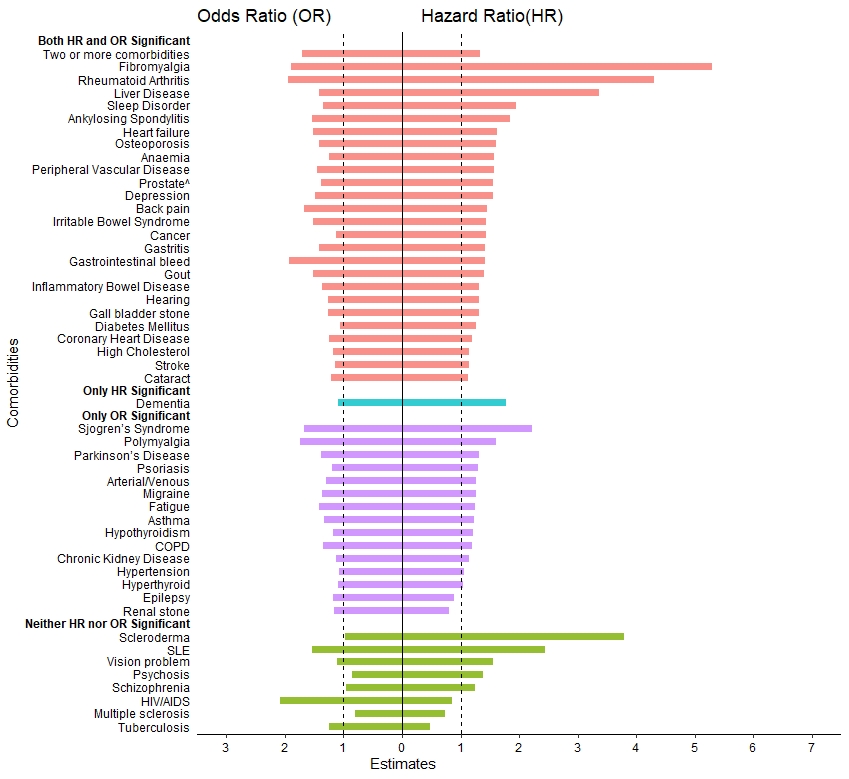


COPD- Chronic obstructive pulmonary diseases; SLE- Systemic lupus erythematous; ***p <0.05; ^Benign prostate hypertrophy -Only men

Red: Both HR and OR significant; Blue: Only HR significant; Purple: Only OR significant; Green: Neither HR nor OR significant

Supplementary Figure S2.3: Comparison of the adjusted hazard ratios comparing the analyses for “OA without any comorbidity” at index date and “OA without the specific comorbidity” at the index date with respective matched controls


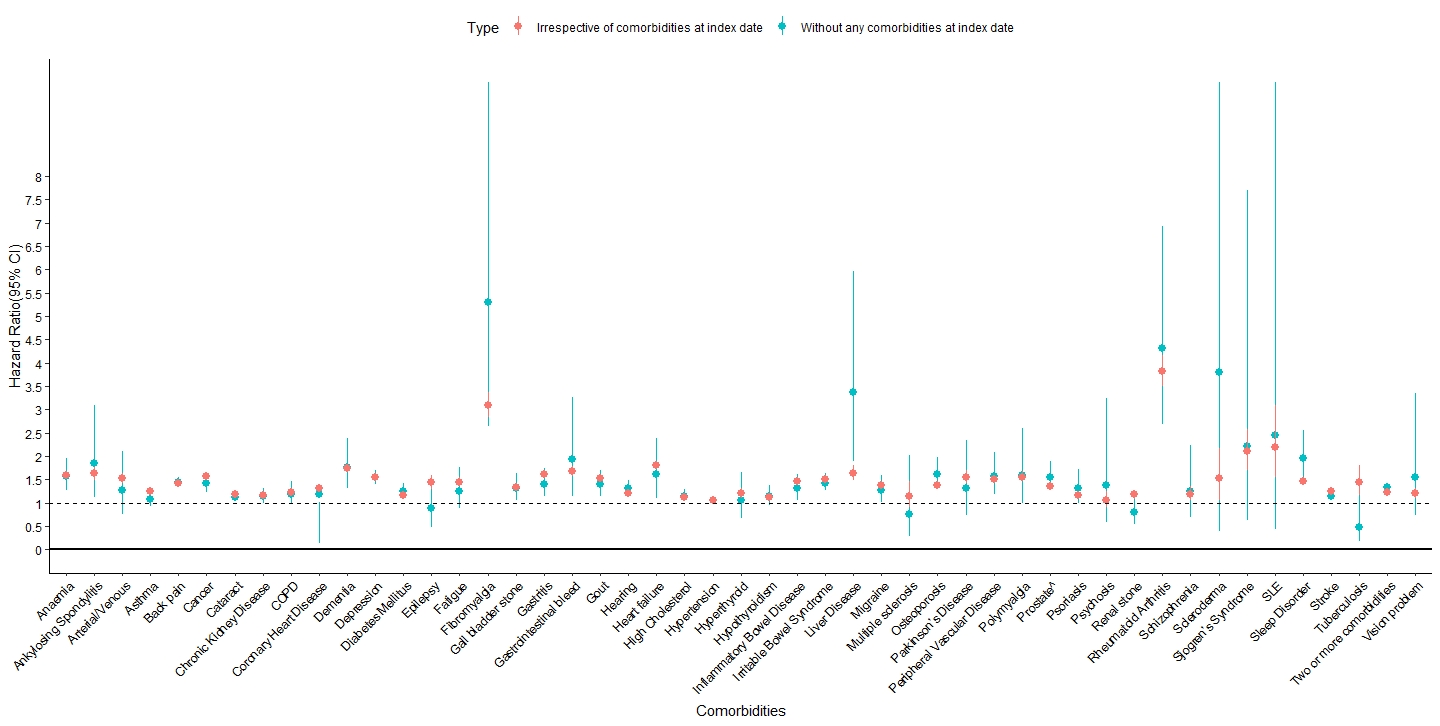


COPD- Chronic Obstructive Pulmonary Disease; SLE- Systemic Lupus Erythematous

^Benign prostate hypertrophy -Only men
